# A novel prognostic 7-methylguanosine signature reflects immune microenvironment and alternative splicing in glioma based on multi-omics analysis

**DOI:** 10.3389/fcell.2022.902394

**Published:** 2022-08-10

**Authors:** Zihan Wang, Zhiwei Zhong, Zehua Jiang, Zepeng Chen, Yuequn Chen, Yimin Xu

**Affiliations:** ^1^ Department of Neurosurgery, The First Affiliated Hospital of Shantou University Medical College, Shantou, China; ^2^ Shantou University Medical College, Shantou, China; ^3^ Joint Shantou International Eye Center, Shantou University and the Chinese University of Hong Kong, Shantou, China; ^4^ School of Medical Sciences, Edith Cowan University, Joondalup, WA, Australia

**Keywords:** glioma, 7-methylguanosine, tumor microenvironment, molecular signature, alternative splicing, multi-omics, spatial transcriptome

## Abstract

Glioma is the most common type of central nervous system tumor with increasing incidence. 7-methylguanosine (m7G) is one of the diverse RNA modifications that is known to regulate RNA metabolism and its dysregulation was associated with various cancers. However, the expression pattern of m7G regulators and their roles in regulating tumor immune microenvironments (TIMEs) as well as alternative splicing events (ASEs) in glioma has not been reported. In this study, we showed that m7G regulators displayed a close correlation with each other and most of them were differentially expressed between normal and glioma tissues. Two m7G signatures were then constructed to predict the overall survival of both GBM and LGG patients with moderate predictive performance. The risk score calculated from the regression coefficient and expression level of signature genes was proved to be an independent prognostic factor for patients with LGG, thus, a nomogram was established on the risk score and other independent clinical parameters to predict the survival probability of LGG patients. We also investigated the correlation of m7G signatures with TIMEs in terms of immune scores, expression levels of HLA and immune checkpoint genes, immune cell composition, and immune-related functions. While exploring the correlation between signature genes and the ASEs in glioma, we found that EIF4E1B was a key regulator and might play dual roles depending on glioma grade. By incorporating spatial transcriptomic data, we found a cluster of cells featured by high expression of PTN exhibited the highest m7G score and may communicate with adjacent cancer cells *via* SPP1 and PTN signaling pathways. In conclusion, our work brought novel insights into the roles of m7G modification in TIMEs and ASEs in glioma, suggesting that evaluation of m7G in glioma could predict prognosis. Moreover, our data suggested that blocking SPP1 and PTN pathways might be a strategy for combating glioma.

## Introduction

Glioma is one of the most common and devastating tumors accounting for approximately 25.6% of all brain and other central nervous system (CNS) tumors and about 82.4% of all malignant tumors (Ostrom et al., 2020, 2013–2017). Gliomas are classified into lower-grade gliomas (LGG) consisting of WHO grade 2 and 3 gliomas and glioblastomas (GBM) belonging to WHO grade 4 gliomas. Despite the integration of treatment modalities such as surgical resection, radiotherapy, and chemotherapy, the clinical outcome of glioma patients is still far from satisfactory (Ho et al., 2014). Thus, discovering new therapeutic targets and prognostic markers for glioma is of great importance.

Diverse RNA modifications play critical roles in RNA metabolisms including pre-mRNA splicing, nuclear exportation, transcript stabilization, and translation initiation, and also take part in the regulation of tumorigenesis (Shi et al., 2020). Following transcription, RNA can undergo more than 170 chemically distinct types of modifications (Wiener and Schwartz, 2021), and m7G is one of these modifications that exist in the internal site of tRNA, rRNA (Rong et al., 2021), and the 5′ cap of mRNA (Malbec et al., 2019). During the past few years, internal m7G sites within mRNA and miRNA were also identified (Pandolfini et al., 2019; Zhang et al., 2019). m7G modification of tRNA occurs most frequently at position 46 in the variable region, stabilizing tRNA via base pair with C13-G22 in the three-dimensional core (Tomikawa, 2018). The yeast small ribosomal subunit (SSU) rRNA and bacterial SSU and large ribosomal subunit (LSU) rRNA are also m7G-modified (Rong et al., 2021). In mRNAs, m7G modification at the 5′ cap regulates their export, translation, and splicing. Besides, METTL1 was identified as a methyltransferase that mediates m7G modification within a subset of mRNA and miRNA that can affect mRNA translation and miRNA maturation respectively (Pandolfini et al., 2019; Zhang et al., 2019). The dysregulation of m7G RNA modification was reported to be associated with various cancers. For example, METTL1/WDR4 mediated m7G tRNA modification increased the translation of oncogenic mRNA with a higher frequency of m7G tRNA-decoded codons, promoting the progression of lung cancer (Ma et al., 2021) and intrahepatic cholangiocarcinoma (Dai et al., 2021).

In recent years, the prognostic value of RNA modification in glioma has been extensively studied. For example, m6A RNA methylation regulator-based risk models were constructed for the prediction of prognosis in astrocytoma (Guo F. et al., 2022), low-grade gliomas (Liu et al., 2021; Zheng et al., 2021), glioblastomas (Cai et al., 2021), and glioma (Guan et al., 2021). However, the expression pattern and prognostic value of genes related to m7G, a newly discovered form of RNA modification, remains unknown in glioma.

In the present study, we characterized the expression pattern of m7G regulators and analyzed their association with TIMEs. Additionally, we constructed m7G signatures and evaluated their role in predicting prognosis. We also investigated the regulatory effect of m7G in alternative splicing.

## 2 Material and methods

### Data extraction

RNA sequencing (RNA-seq) data of 698 glioma samples (169 GBM and 529 LGG) and 5 normal controls were obtained from The Cancer Genome Atlas (TCGA) database. After removing samples of recurrent tumor, 667 primary glioma samples (156 GBM and 511 LGG) were left for analyzing the expression profile of m7G regulators. Among them, 659 patients (153 GBM and 506 LGG) with complete follow-up information were further selected for analyzing the correlation between m7G regulators and prognosis, TIMEs, and ASEs. The corresponding clinical information was downloaded from UCSC Xena (http://xena.ucsc.edu/). The clinical characteristics of patients involved in this study were summarized in Table 1. Proteomic data of 100 GBM patients and 10 normal controls were downloaded from the CPTAC database (PDC000204). The protein level of signature genes was obtained online from the Human Protein Atlas (HPA, www.porteinatlas.org). 33 m7G-related genes were collected from the literature (Tomikawa, 2018) and the Gene Set Enrichment Analysis website (http://www.gsea-msigdb.org). The m7G-related genes were presented in Supplementary Table S1

**TABLE 1:** Clinical characteristics of patients involved in this study.

Characteristics	TCGA-GBM (*n* = 153)	TCGA-LGG (*n* = 506)
Age(years)		
<65	96	471
≧65	57	35
Gender		
Male	99	280
Female	54	226
Race		
Asian	5	8
American Indian	0	1
Black or Africa American	10	21
White	137	466
Unknown	1	10
WHO grade		
WHO2	0	245
WHO3	0	260
WHO4	153	0
Unknown	0	1
Vital status		
Alive	29	380
Dead	122	125
Unknown	2	1
Radiotherapy		
Yes	123	273
No	21	167
Unknown	9	66
Person tumor status		
With tumor	123	237
Tumor free	13	167
Unknown	17	102

### The expression profile of m7G regulators

The correlation network and correlation heatmap of m7G regulators were constructed via the “igraph” and “corrplot” R packages respectively. The expression levels of m7G regulators among normal tissue, GBM, and LGG were compared by the Kruskal-Wallis test. The Wilcox test was applied to compare the difference in the expression level of m7G regulators between any two of the three groups. A *p*-value of less than 0.05 was considered statistically significant.

### Construction of prognostic m7G signature

The univariate Cox regression analysis was performed to screen prognostic m7G regulators (*p*-values<0.05) and the multivariate Cox regression was applied to construct prognostic m7G signatures for both GBM and LGG cohorts. The risk score was then calculated for each patient as follow:

Risk score=β(Gene1)∗exp(Gene1)+β(Gene2)∗exp(Gene2)+……β(Gene n)∗exp(Gene n) (β: coefficients; exp: gene expression level).

Patients were then further stratified into low- and high-risk groups according to the median risk score. The survival difference between two risk groups was assessed by the Kaplan-Meier method via the “survival” and “survminer” R packages. The accuracy of the m7G signature in predicting prognosis was evaluated by the ROC curve and the area under the curve (AUC). Univariate and multivariate Cox regression analyses were employed to examine whether the m7G signature was an independent risk factor for OS.

### Building of prognostic nomogram

The risk score and available clinicopathological parameters with possible prognostic values were subjected to the univariate Cox regression and multivariate Cox regression analyses to identify independent prognostic factors. Based on independent prognostic factors, a nomogram was constructed to predict the survival probability by using the “rms” R package. The discrimination of the nomogram was assessed by calculating the concordance index (C-index). The relationship between the predicted and observed risk for the outcomes of the nomogram was graphically displayed as calibration plots.

### Analysis of immune microenvironment

The expression level of HLA-related genes and immune checkpoint genes were extracted from the RNA sequencing data. The Estimation of Stromal and Immune cells in Malignant Tumor tissues using Expression data (ESTIMATE) algorithm was used to estimate the immune score and stromal score. The CIBERSORT algorithm was used to estimate the infiltration level of immune cells. The single-sample gene set enrichment analysis (ssGSEA) was conducted by the “gsva” R package to quantify the infiltrating immune cells and the immune-related functions of each sample.

### Analysis of alternative splicing events

The imputed Percent Spliced In value (PSI) of 7 alternative splice event (ASE) types (AA: Alternate Acceptors; AD: Alternate Donors; ES: Exon Skip; RI: Retained Intron; AP: Alternate Promoters; AT: Alternate Terminators; ME: Mutually Exclusive Exons) for patients in TCGA-GBM and TCGA-LGG cohorts were downloaded from the TCGA SpliceSeq database (https://bioinformatics.mdanderson.org/TCGASpliceSeq/). The univariate Cox model was performed to identify OS-related splice events (OS-SEs), which were then presented in UpSet plots (“UpSetR” R package). Moreover, the top 20 OS-SEs of each type of ASEs were shown in bubble plots. Regulatory networks between signature genes and OS-SEs were constructed via Cytoscape (3.9.0) based on Pearson correlation analyses.

### Analysis of spatial gene expression data

Spatial gene expression data of a human glioblastoma case was downloaded from 10xGENOMICS (https://www.10xgenomics.com/) datasets and processed via R packages “hdf5r”, “Seurat” and “CellChat”.

### Statistical analysis

Statistical analyses were performed using R software v 4.1.3. *p*-value <0.05 was considered statistically significant

## 3 Results

### The expression profile of 7-methylguanosine regulators in glioma

**FIGURE 1:**
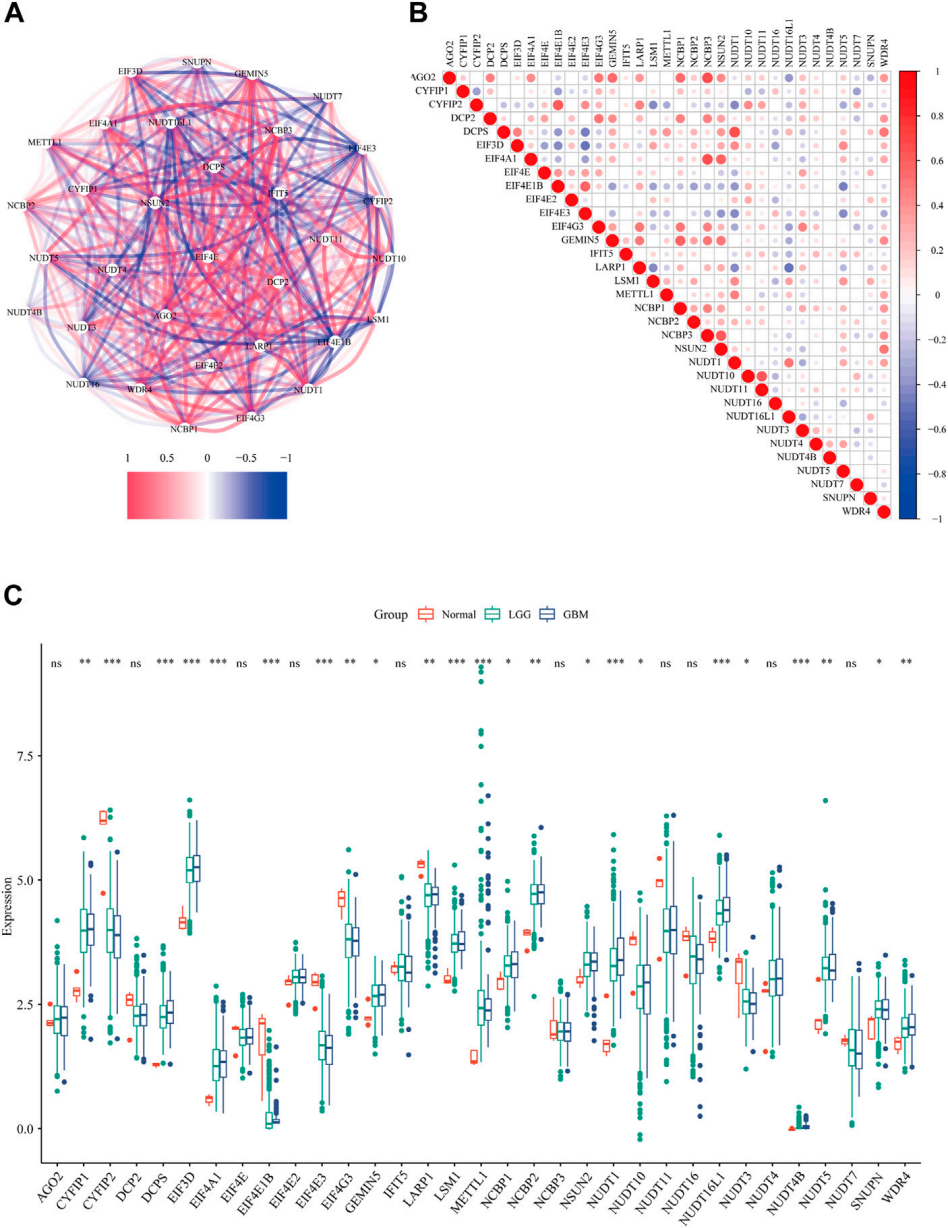
The expression profile of m7G regulators in glioma. **(A)** The correlations network of 33 m7G regulators (red lines represent positive correlation and blue lines represent negative correlation, the darkness of color represents the strength of correlation). **(B)** The correlation heatmap of 33 m7G regulators (red dots represent positive correlation and blue dots represent negative correlation, the darkness of color represents the strength of correlation). **(C)** The expression levels of m7G-related genes among normal tissue, GBM and LGG (Kruskal-Wallis test, *p < 0.05; **p < 0.01; ***p < 0.001)

Firstly, we extracted the expression data of 33 m7G regulators from the TCGA data set. The correlation network (Figure 1A) and the spearman analysis of correlation coefficients (Figure 1B) indicated an intensive connection among them. By comparing the expression level of m7G regulators among normal tissue, GBM and LGG, 23 were found to be differentially expressed (*p* < 0.05). Specifically, 16 genes (CYFIP1, DCPS, EIF3D, EIF4A1, GEMIN5, LSM1, METTL1, NCBP1, NCBP2, NSUN2, NUDT1, NUDT16L1, NUDT4B, NUDT5, SNUPN, and WDR4) were up-regulated while 8 genes (CYFIP2, EIF4E1B, EIF4E3, EIF4G3, LARP1, NUDT10, NUDT3) were down-regulated in tumor tissue (Figure 1C). We noticed that the expression level of most m7G regulators was similar between GBM and LGG except for DCPS, EIF4E1B, NUDT1, and NUDT16L1 (Supplementary Figures S1A–C). Given the fact that GBM has a worse prognosis compared to LGG, the higher level of these 4 m7G regulators in GBM might suggest their contribution to the malignancy of glioma.

### The prognostic value of 7-methylguanosine regulators in glioma

**FIGURE 2:**
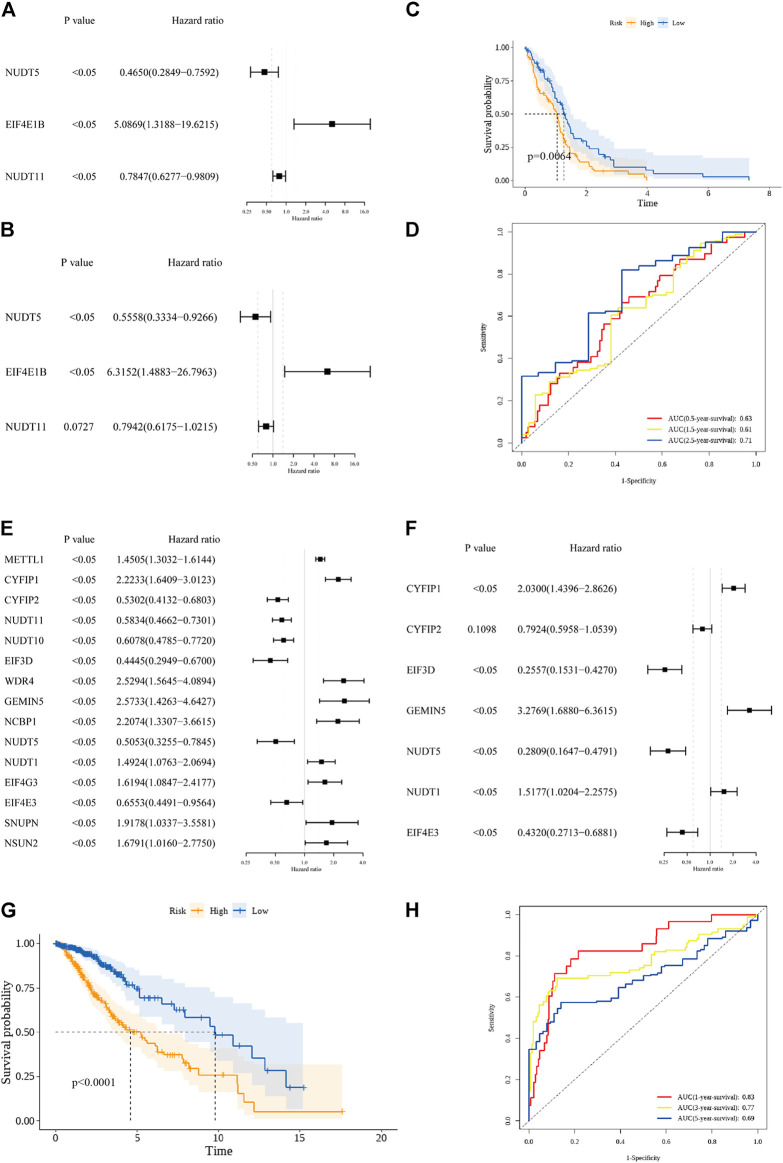
Construction of the prognostic m7G signature. **(A)** 3 prognostic m7G regulators in the GBM cohort were screened by the univariate cox regression analysis. **(B)** 3 m7G regulators were incorporated by the multivariate cox regression analysis to build the prognostic m7G signature in the GBM cohort. **(C)** Kaplan-Meier survival curve for the OS of patients in the high-risk (yellow) group and low-risk group (blue) in the GBM cohort. **(D)** AUC of the time-dependent ROC analysis for evaluating the prognostic performance of the risk score in the GBM cohort. **(E)** 15 prognostic m7G regulators in the LGG cohort were screened by the univariate cox regression analysis. **(F)** 7 m7G regulators were incorporated by the multivariate cox regression analysis to build the prognostic m7G signature in the LGG cohort. **(G)** Kaplan-Meier survival curve for the OS of patients in the high-risk (yellow) group and low-risk group (blue) in the LGG cohort. **(H)** AUC of the time-dependent ROC analysis for evaluating the prognostic performance of the risk score in the LGG cohort.

In the GBM cohort, 3 OS-related m7G regulators (NUDT5, EIF4E1B, and NUDT11) were identified by the univariate Cox regression analysis (*p* < 0.05, Figure 2A). Among them, EIF4E1B was associated with poor prognosis (HR > 1) while NUDT5 and NUDT11 were predictors of favorable outcomes (HRs<1). These 3 genes were further incorporated by the multivariate Cox regression model to construct the prognostic m7G signature for GBM (Figure 2B). The risk scores based on regression coefficient and expression level of genes in the signature were calculated as follow: Risk score = 1.843* exp (EIF4E1B)—0.230* exp (NUDT11)—0.587* exp (NUDT5). By using the median risk score as a cutoff value, 153 GBM patients were subdivided into low- (*n* = 77) and high-risk (*n* = 76) groups. A significant difference in survival probability between the two risk groups was revealed by Kaplan-Meier survival analysis (*p* value < 0.05, Figure 2C). Then, the ROC analysis was performed to evaluate the predictive sensitivity and specificity of our risk model at 0.5, 1.5, and 2.5 years and the corresponding AUC value was 0.63, 0.61, and 0.71 respectively (Figure 2D). In the LGG cohort, 15 prognostic m7G regulators (METTL1, CYFIP1, CYFIP2, NUDT11, NUDT10, EIF3D, WDR4, GEMIN5, NCBP1, NUDT5, NUDT1, EIF4G3, EIF4E3, SNUPN, and NSUN2) were screened by the univariate Cox regression analysis (*p* < 0.05, Figure 2E). Among them, 9 genes (METTL1, CYFIP1, WDR4, GEMIN5, NCBP1, NUDT1, EIF4G3, SNUPN, and NSUN2) were risk genes with HRs>1 while the other 6 genes (CYFIP2, NUDT11, NUDT10, EIF3D, NUDT5, and EIF4E3) were favorable genes with HRs<1. Subsequently, 7 genes were incorporated by the multivariate Cox regression model to construct the prognostic m7G signature (Figure 2F). Similarly, the risk scores for each LGG patient were calculated as follow: Risk score = 0.708* exp (CYFIP1)—0.233* exp (CYFIP2) – 1.364* exp (EIF3D)—0.839* exp (EIF4E3) + 1.187* exp (GEMIN5) + 0.417* exp (NUDT1)—1.270* exp (NUDT5). Based on the median risk score, 506 LGG patients were evenly assigned into the low- and high-risk groups with significant survival difference (Figure 2G). The AUC value of the ROC curve at 1, 3, and 5 years were 0.83, 0.77, and 0.69 respectively (Figure 2H). These data suggested that prognostic signatures constructed on m7G regulators can predict the overall survival of both GBM and LGG patients with moderate performance.

To better characterize the expression of m7G signature genes, mRNA expression data of the GBM cohort (169 GBM cases and 5 normal controls) were extracted from the TCGA database, and proteomic data of 100 GBM cases and 10 normal controls were downloaded from the CPTAC database (PDC000204) and the immunohistochemical data of GBM cases and normal controls were obtained online from the Human Protein Atlas. As illustrated in Supplementary Figure S2.1–S2.3, the mRNA level and protein levels of CYFIP1, EIF3D, GEMIN5, and NUDT1 were higher in GBM samples compared with normal controls, while the mRNA and protein levels of CYFIP2 were lower in GBM samples. As for EIF4E3, NUDT5, and NUDT11, though the difference in mRNA levels was observed between normal and GBM tissues, their protein levels were similar. The mRNA level of EIF4E1B was higher in normal tissue compared to GBM, however, mass spectrometry failed to detect its protein product, constantly, immunohistochemical staining of EIF4E1B was negative in both normal and GBM tissues.

### The independent prognostic value of the m7G signatures

**FIGURE 3:**
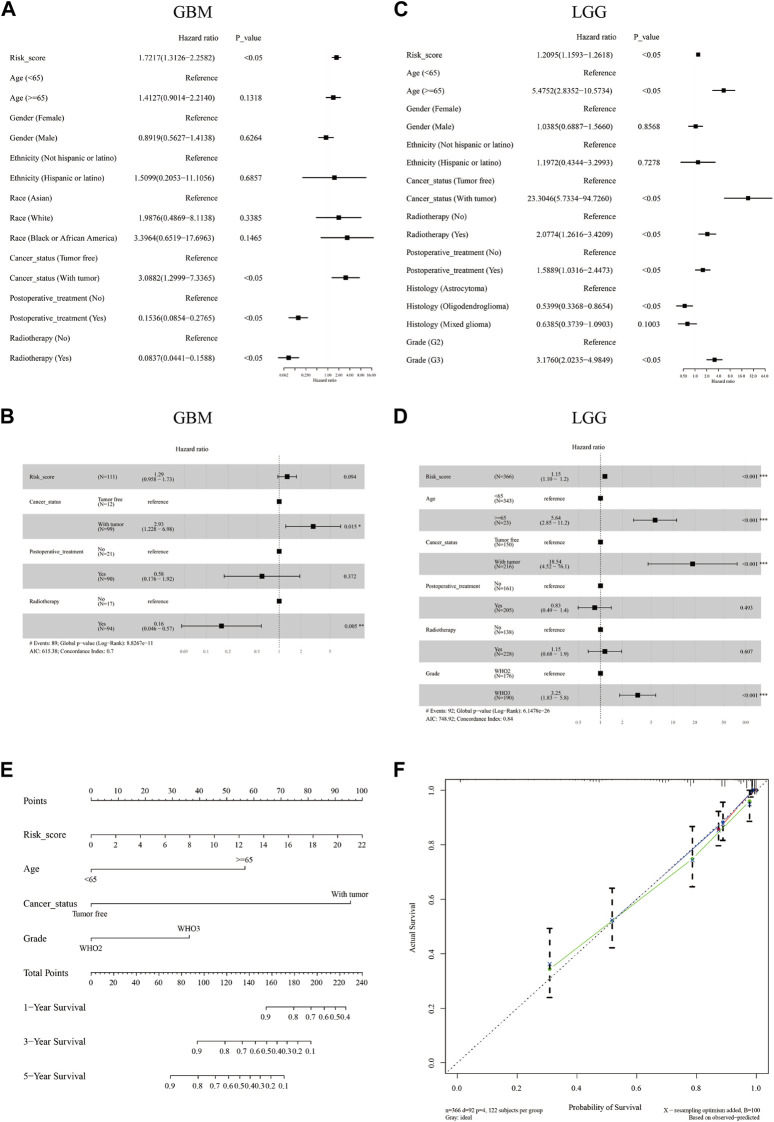
The independent prognostic value of the m7G signature and the construction of a predictive nomogram. Univariate **(A,C)** and multivariate **(B,D)** Cox regression analysis of the risk score and clinical factors in the GBM cohort **(A,B)** and the LGG cohort **(C,D)**. A nomogram was constructed based on independent predictive factors for predicting the 1-, 3-, and 5- year survival probability of LGG patients **(E)**. Calibration plot for validating the accuracy of the nomogram **(F)**.

Furthermore, univariate and multivariate Cox regression analyses were performed in both cohorts to evaluate whether the risk score could be an independent prognostic predictor for OS in glioma patients. Our results indicated that in the GBM cohort, the risk score was not an independent prognostic predictor with HRs (95% CI) of 1.7217 (1.3126–2.2582) and 1.29 (0.958–1.73) in the univariate and multivariate Cox regression analyses respectively (Figures 3A,B). In the LGG cohort, the risk score was proved to be an independent poor prognosis predictor with HRs (95% CI) of 1.2095 (1.593–1.2618) and 1.15 (1.10–1.2) in the univariate and multivariate Cox regression analyses respectively (Figures 3C,D). Based on the independent prognostic parameters (risk score, age, cancer status, and WHO grade), we constructed a nomogram to predict the 1-, 3-, and 5-year survival probabilities of LGG patients (Figure 3E). The C-index of the nomogram was 0.837. The 1-, 3-, and 5-year calibration curves showed a favorable consensus between the survival predicted by nomogram and the actual survival (Figure 3F)

### The relationship between m7G signature and the immune microenvironment in glioma

**FIGURE 4:**
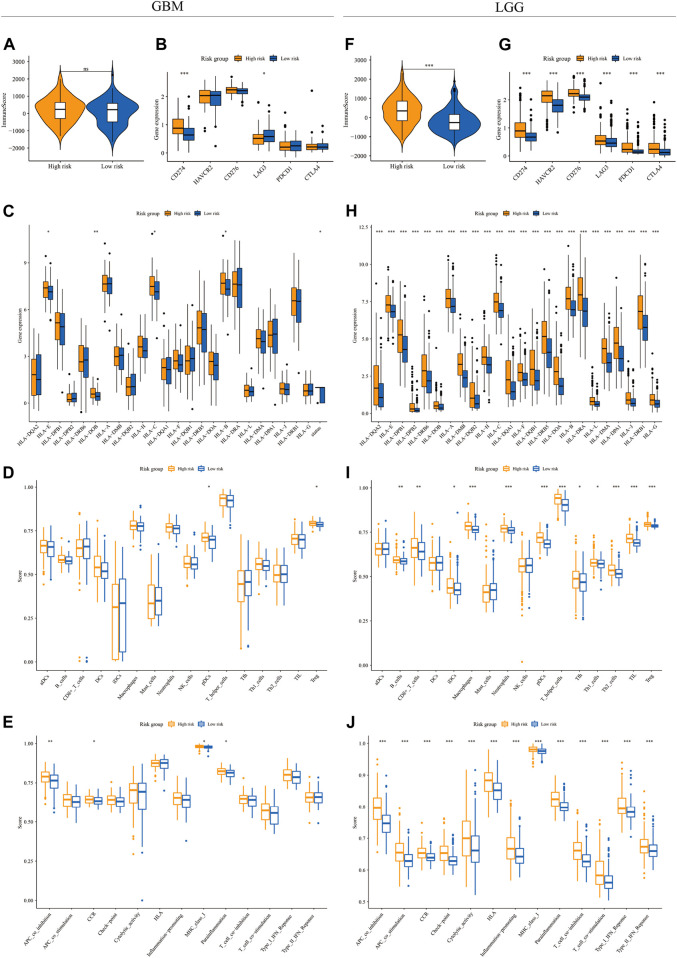
The immune landscape in GBM and LGG subgroups. The violin plot and boxplots showed the immune score **(A,F)**, expression level of immune checkpoint genes **(B,G)** and HLA genes **(C,H)**, infiltration level of various immune cell types **(D,I)**, and activity of immune functions **(E,J)** between high-risk (yellow) and low-risk groups (blue) in the GBM **(A–E)** and LGG **(F–J)** cohorts (Wilcox test, *p < 0.05; **p < 0.01; ***p < 0.001)

To depict the immune landscape in two risk groups stratified by m7G signatures in both GBM and LGG cohorts, we first calculated the immune score from transcriptomic data using the ESTIMATE algorithm. The immune score was similar between the two risk groups in the GBM cohort (Figure 4A) but significantly higher in the high-risk group in the LGG cohort (Figure 4F). Next, we compared the expression of the immune checkpoint genes between two risk groups. In the GBM cohort, up-regulation of CD274 and down-regulation of LAG3 were observed in the high-risk group compared to the low-risk group (Figure 4B). In the LGG cohort, the expression level of all immune checkpoint genes (CD274, HAVCR2, CD276, LAG3, PDCD1, and CTLA4) increased significantly in the high-risk group (Figure 4G). We also explored the expression of HLA-related genes in both cohorts. The result showed a significantly higher expression level of HLA-E, HLA-DOB, HLA-C, and HLA-B in the high-risk group of the GBM cohort (Figure 4C). In the LGG cohort, the expression levels of all HLA genes were higher in the high-risk group (Figure 4H).

**FIGURE 5:**
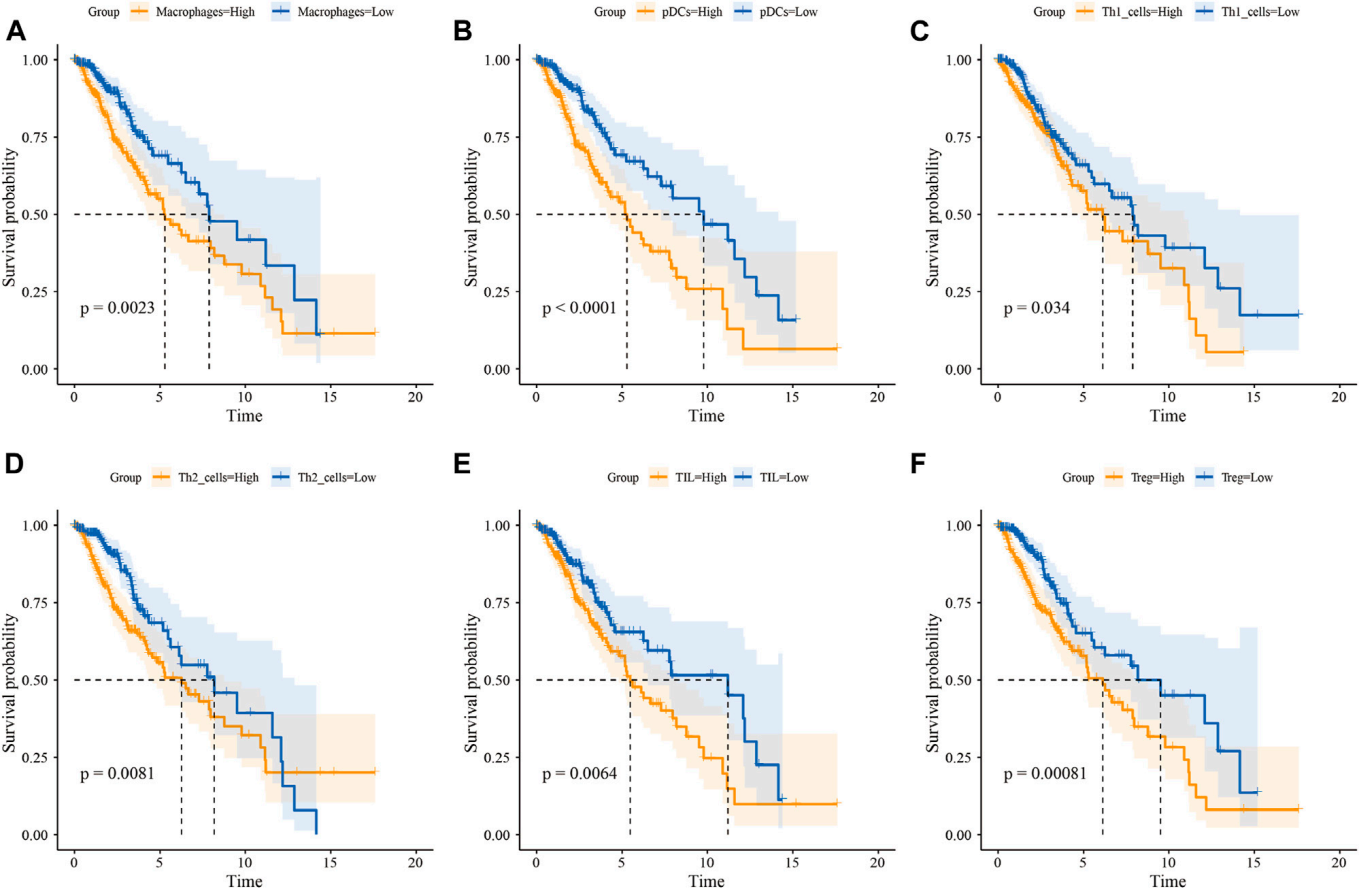
Infiltration level of immune cells predicts OS of LGG patients. Kaplan-Meier survival curve for the OS of LGG patients subdivided by macrophage **(A)**, pDC **(B)**, Th1 cell **(C)**, Th2 cell **(D)**, TIL **(E),** and Treg **(F)** infiltration level (yellow lines represent high infiltration group and blue lines represent low infiltration group)

We then quantified the infiltration level of 16 immune cells and the activity of 13 immune-related pathways between the low- and high-risk groups *via* ssGSEA. We found that tumor tissues from GBM patients in the high-risk group had a significantly higher infiltration level of pDCs and Tregs (Figure 4D), however, neither of them were associated with patient outcomes (Supplementary Figures S3A,B). In the LGG cohort, tumor tissues from the high-risk group were predicted to contain a higher level of many immune cell types, including B cells, CD8^+^ T cells, iDCs, macrophages, neutrophils, pDCs, T helper cells, Tfh, Th1 cells, Th2 cells, TIL, and Treg (Figure 4I). Among them, the higher infiltration level of macrophages, pDCs, Th1 cells, Th2 cells, TIL, and Treg was associated with poor clinical outcomes in the LGG cohort (Figure 5 and Supplementary Figures S3C–H). In terms of immune pathways, the activity of pathways relating to APC co-inhibition, CCR, MHC class1, and para-inflammation were predicted to be higher in the high-risk group in the GBM cohort (Figure 4E). In the LGG cohort, all the 13 immune pathways assessed showed higher activity in the high-risk group than in the low-risk group (Figure 4J).

### The relationship between m7G regulators and alternative splicing in glioma

**FIGURE 6:**
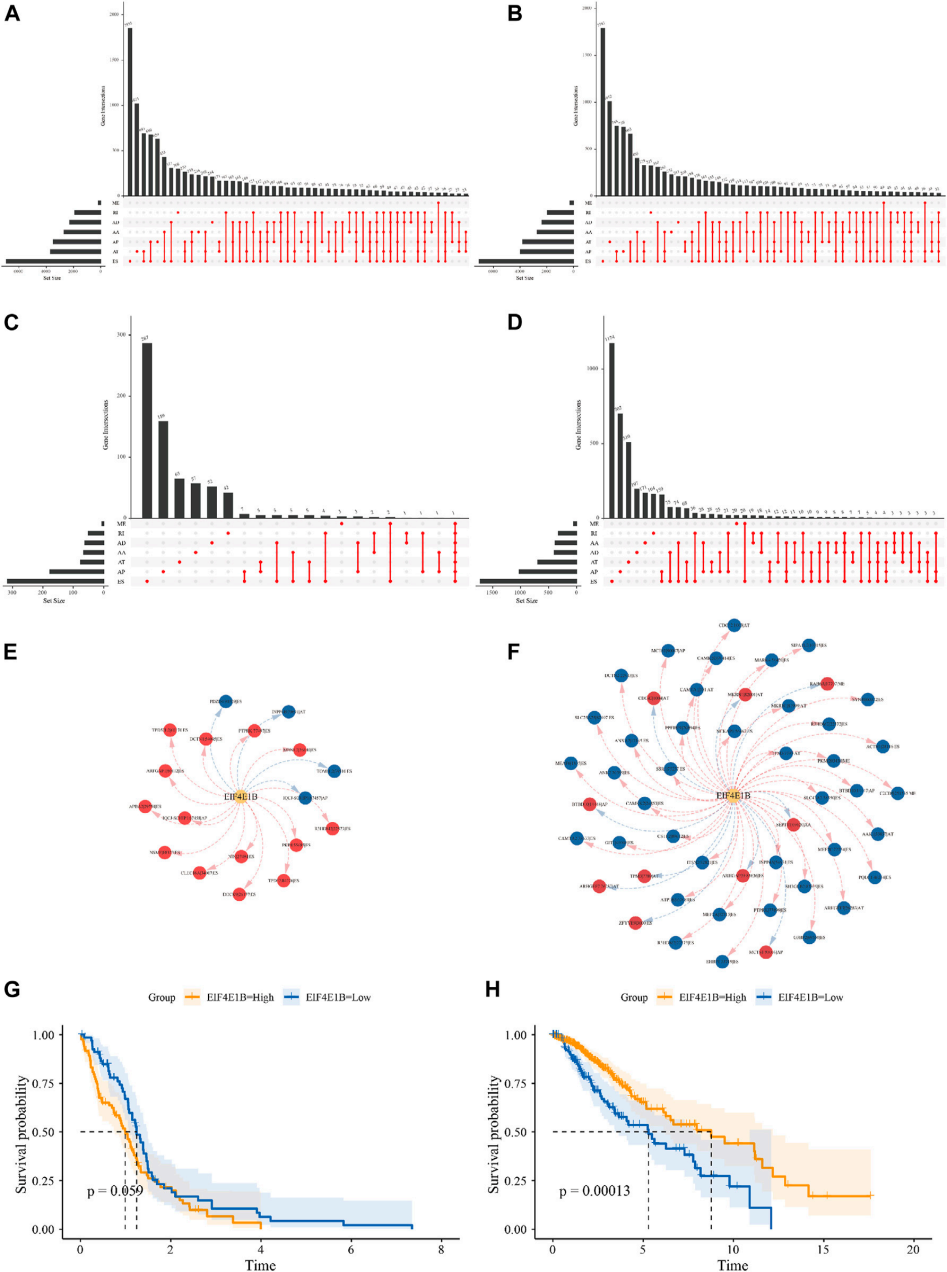
Relationship between m7G regulators and alternative splicing in glioma The Upset plot of seven types of ASEs in the GBM **(A)** and LGG **(B)** cohort derived from the TCGA database. The Upset plot of seven types of ASEs associated with the overall survival of GBM **(C)** and LGG **(D)** patients. The splicing correlation network between EIF4E1B and OS-SEs in GBM **(E)** and LGG **(F)** (risk SEs with HRs>1 were represented by red dots, favorable SEs with HRs<1 were represented by blue dots. Red arrows indicate positive correlation while blue arrows indicate negative correlation). The Kaplan-Meier survival curves showed different survival probabilities in GBM **(G)** and LGG **(H)** patients stratified by EIF4E1B expression level. (Yellow lines represent the high expression group and blue lines represent the low expression group).

In mRNA, m7G modification at the 5′ cap was implied to regulate RNA splicing, to explore the possible regulatory effect of m7G regulators on alternative splicing in glioma, PSI scores of 7 types of ASEs for 154 GBM patients and 510 LGG patients were obtained from the TCGA SpliceSeq database. Among them 45610 ASEs in 10434 genes were identified in GBM cases, including 3827 AAs (2,684 genes), 3269 ADs (2,270 genes), 8686 APs (3,476 genes), 8456 ATs (3,696 genes), 18360 ESs (6,935 genes), 184 MEs (181 genes) and 2828 RIs (1,898 genes) (Figure 6A). A total number of 48050 ASEs in 10788 genes were recognized in LGG cases, containing 3876 AAs (2,720 genes), 3351 ADs (2,353 genes), 9964 APs (3,976 genes), 8718 ATs (3,810 genes), 18931 ESs (7,076 genes), 273 MEs (262 genes) and 2937 RIs (1,971 genes) (Figure 6B). These results indicated that one gene can undergo several types of splicing events and the predominant splicing pattern in both cohorts was ES. Subsequently, univariate Cox analysis revealed 975 and 7,213 ASEs to be correlated with OS of GBM and LGG patients respectively (Figures 6C–D). We showed the top 20 OS-ASEs of the 7 types of splicing patterns as bubble plots (Supplementary Figure S4–S5) and the most significant OS-SE was the ZNF280D-30765-AP for GBM and the UGP2-53745-AP for LGG. Next, we used Pearson correlation analysis to explore the underlying connection between 23 differentially expressed m7G regulators and OS-SEs, those with *p* < 0.001 and |correlation coefficient|>0.6 were kept for regulatory network construction. In the GBM cohort, EIF4E1B was the only signature gene that correlated with OS-SEs (Figure 6E, Supplementary Table S2). Specifically, EIF4E1B was positively correlated with risk ASEs (HR > 1) and negatively correlated with protective ASEs (HR < 1). In the LGG cohort, CYFIP1, CYFIP2, EIF3D, EIF4A1, EIF4E1B, EIF4E3, EIF4G3, GEMIN5, NSUN2, NUDT1, NUDT10, and NUDT16L1 were predicted to be associated with many OS-SEs (Supplementary Figure S6, Supplementary Table S3). Interestingly, EIF4E1B was positively correlated with almost all protective ASEs and negatively correlated with risk ASEs in the LGG cohort (Figure 6F, Supplementary Table S3). Indicating that EIF4E1B might be a risk factor in GBM while a protective factor in LGG. To test this notion, we subdivided patients into EIF4E1B-high and EIF4E1B-low expression groups based on cutoff values determined by the “surv_cutpoint” function of R package “survminer” (0.1252297 for GBM and 0.02686604 for LGG) and compared the survival difference by the Kaplan-Meier method. It is revealed that though statistically not significant, GBM patients in EIF4E1B high expression group tend to have worse clinical outcomes (Figure 6G), however, in the LGG cohort, high EIF4E1B expression was significantly associated with better survival probability (Figure 6H).

### Intra-tumoral heterogeneity in the context of m7G regulators

**FIGURE 7:**
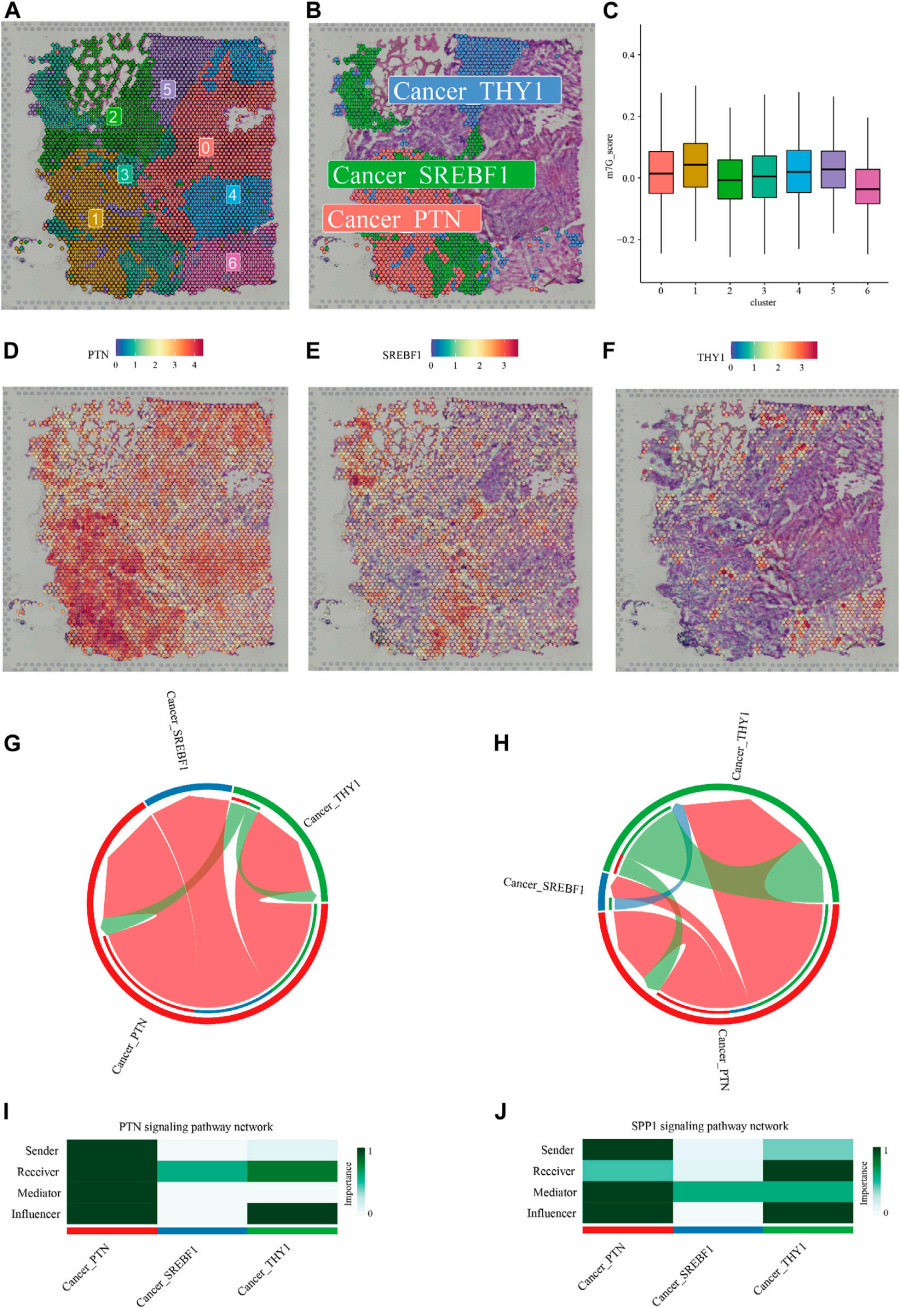
Intra-tumoral heterogeneity in the context of m7G regulators expression. **(A)** Hematoxylin and eosin **(H,E)** staining of tissue section labeled with cluster information. **(B)** The Spatial relationship between a cluster of cells with the highest m7G score (Cancer_PTN) and two clusters of cells (Cancer_SREBF1, and Cancer_THY1) in its proximity. **(C)** Violin plots showed the m7G score of 7 clusters of cells identified by unsupervised clustering. **(D–F)** Cell markers of cluster 1, cluster 3, and cluster 5. Cell-cell communications via the SPP1 **(G,I)** and PTN **(H,J)** pathway predicted by the “CellChat” R package were shown.

A distinct pathological feature of glioma, especially GBM is the high degree of inter- and intra-tumoral heterogeneity (Gularyan et al., 2020). The above exploration of m7G regulators' expression profile and their correlation with TIMEs as well as alternative splicing events described the inter-tumoral heterogeneity in the context of RNA m7G modification. To further investigate the intra-tumoral heterogeneity of m7G modification within an individual tumor, we analyzed spatial gene expression data of a case of GBM obtained from 10XGENOMICS datasets. Firstly, using resolution determined by the “clustree” R package (resolution = 0.2, Supplementary Figure S7), 7 clusters of cells were identified by unsupervised clustering (Figure 7A), and the top 10 highly expressed genes were recognized as maker genes of a given cluster (Supplementary Table S4). We then establish a m7G score for each cell based on 16 m7G regulators whose expression was significantly higher in GBM compared to normal tissue. The violin plot showed that cluster 1, marked by high expression of PTN, exhibited the highest m7G score (Figures 7C,D). We noticed that cells from cluster 3 (characterized by high expression of gene SREBF1, Figure 7E) and cluster 5 (characterized by high expression of THY1, Figure 7F) embedded with cluster 1 spatially, implying possible intercellular crosstalk. We then predict the communication among these 3 cell types using the “CellChat” R package (Figure S8). As shown in Figures 7G–J, the two most significant communicating pathways were SPP1 and PTN.

## 4 Discussion

Among central nervous system tumors in adults, glioma represents the most common type, and the incidence of diffuse glioma is about 1000,000 cases per year (Molinaro et al., 2019). Epidemiology data indicated an increased incidence of brain tumors during the past decades (Grech et al., 2020), arguing for the discovery of novel therapeutic targets and prognostic markers.

Methylation signature has been reported to be associated with survival in glioma, for example, a two-CpG site DNA methylation signature was constructed to predict the prognosis of lower-grade glioma with high accuracy (Guo W. et al., 2022). Additionally, a DNA methylation-driven genes signature was established for risk stratification in GBM (Yu et al., 2021). m6A RNA methylation was reported to regulate the tumorigenesis of glioma. A recent study comprehensively investigated the m6A regulators in glioma revealed that m6A-readers were more significantly involved in the stability, translation efficiency, alternative splicing, and localization of mRNA in LGG than in GBM (Zhao et al., 2021). Besides, prognostic m6A RNA methylation signatures have been constructed in CNS tumors (Cai et al., 2021; Guan et al., 2021; Liu et al., 2021; Zheng et al., 2021; Guo F. et al., 2022). However, the prognostic value of other methylation signatures like 7-methylguanosine is not yet determined despite its involvement in cancer development being validated by recent studies.

In this study, we first characterized the expression pattern of m7G regulators based on expression data from TCGA. Close connections were observed among 33 m7G regulators and 23 out of them were differentially expressed between normal and glioma tissues. Univariate and multivariate Cox analyses were subsequently performed to construct m7G signatures to predict the overall survival probability in both GBM and LGG. Here, we discussed the specific roles and clinical relevance of m7G regulators in the signatures as follows:

Translation initiation is a critical step in controlling protein expression levels. In most cases, translation initiation in eukaryotes is mediated by the assembly of the eukaryotic translation initiation complex eIF4F to the 5’ m7G cap (Hinnebusch, 2014). eIF4F comprises the cap-binding protein eIF4E, eIF4G, and the RNA helicase eIF4A (Hinnebusch, 2014). EIF4E1B gene was thought to arise in tetrapoda as a result of the ancestral EIF4E locus duplication. (Evsikov and Marín de Evsikova, 2009). In contrast to the ubiquitously expressed eIF4E protein, eIF4E1b expression is confined to ovaries, oocytes, and early embryos in mice, zebrafish, and Xenopus (Kubacka et al., 2015). Though eIF4E1b is a paralog of eIF4E with conserved cap-binding residues, its cap-binding affinity is 3-fold less well than eIF4E (Kropiwnicka et al., 2015; Kubacka et al., 2015) and hence promote translation initiation at a lower rate compared with eIF4E (Patrick et al., 2014). It is reported that eIF4E1b is a component of the CPEB (cytoplasmic polyadenylation element-binding protein) mRNP repressor complex, which inhibits protein synthesis in Xenopus oocytes (Minshall et al., 2007; Standart and Minshall, 2008). However, the expression and function of EIF4E1B gene and eIF4E1b protein have not been reported in humans, our results showed that the mRNA level of EIF4E1B decreased in glioma tissue, indicating possible anti-tumor effects. We also revealed that the expression of EIF4E1B was positively correlated with risk ASEs and negatively correlated with protective ASEs in GBM. Oppositely, it is positively correlated with protective ASEs and negatively correlated with risk ASEs in LGG. These findings suggested that EIF4E1B might perform dual roles depending on glioma grade. However, immunohistochemical staining and proteomic technique failed to detect the eIF4E1b protein in both normal cortex and glioma tissues. There could be two explanations that remained to be further validated by experiments, one is that eIF4E1b was degraded rapidly after functioning and the other one is that EIF4E1B functions in form of RNA rather than protein.

The assembly of eIF4F on the 5′ terminal m7G cap is often regulated by the 4E binding protein (4E-BPs), which interferes with the eIF4E-eIFG interaction. The cytoplasmic fragile x mental retardation protein-interacting protein 1 (CYFIP1) functions as a 4E-BP to interact with the eIF4F and the Fragile X mental retardation protein (FMRP). In the brain, the eIF4E-CYFIP1-FMRP complex is present at synapses, repressing protein translation (Napoli et al., 2008). CYFIP1 was reported to be associated with brain diseases (Schenck et al., 2001) such as schizophrenia (Zhao et al., 2013) and autism spectrum disorders (Waltes et al., 2014). CYFIP2 is another member of the CYFIP family that was associated with Alzheimer’s disease (Tiwari et al., 2016), intellectual disability (Zweier et al., 2019), and early-onset epileptic encephalopathy (Nakashima et al., 2018). Though being recognized as an RNA 7-methylguanosine cap-binding protein, the specific role of CYFIP2 in m7G modification has not yet been determined.

Alternative to eIF4F mediated translation initiation, EIF3 can bind to the m7G cap of mRNA to promote the translation of specific mRNA containing structures that prevent the actions of eIF4F (Cate, 2017). EIF3D is a subunit of EIF3 that facilitates the cap-dependent translation of approximately 20% of mRNA (de la Parra et al., 2018). EIF3D was widely studied in cancer and was known to promote the progression of cervical cancer (Zhong and Lan, 2022, 78), gallbladder cancer (Zhang et al., 2017), and renal cell carcinoma (Pan et al., 2016; Huang et al., 2019). Besides, EIF3D might serve as an independent poor prognostic marker in lung adenocarcinoma (Wang et al., 2019)

Gemin5 is a multitasking protein that was reported to crosstalk with the translation machinery in different ways (Piñeiro et al., 2015). Gemin5 can interact with the eIF4E (Fierro-Monti et al., 2006) or displays m7G cap-binding capacity (Bradrick and Gromeier, 2009) to regulate cap-dependent translation, besides, it can also interact with the internal ribosome entry site (IRES) of foot-and-mouth disease virus (FMDV) and hepatitis C virus (HCV) to regulate IRES-driven translation (Pacheco et al., 2009).

NUDT1, NUDT5 and NUDT11 are proteins with m7G(5′)pppN-diphosphatase activity that catalyze the reaction 7-methylguanosine 5′-triphospho-5′-polynucleotide + H2O = 7-methylguanosine 5′-phosphate + polynucleotide. Hydrolysis of oxidized DNA precursors by NUDT1(MTH1) is a way to prevent mutagenesis (Nakabeppu et al., 2017). Cancer cells usually exhibit unregulated ROS production that might oxidize DNA as well as free dNTP. It was shown that cancer cells require NUDT1 to sanitize oxidized dNTP, preventing DNA damage and cell death caused by the incorporation of oxidized dNTP. In the same study, NUDT1 inhibition by small molecules was validated to suppress cancer growth by accumulating oxidative damage (Gad et al., 2014). In addition to NUDT1, NUDT5 is also capable of degrading oxidized DNA precursors, avoiding the occurrence of mutations (Ishibashi et al., 2003). Elevated NUDT5 level has been reported as a poor prognostic marker of breast cancer (Tong et al., 2021), non-small cell lung cancer (Li et al., 2021), esophageal squamous cell carcinoma (Wang et al., 2020), and clear cell renal cell carcinoma (Wang et al., 2017). NUDT11 might associate with encephalitozoonosis (Desoubeaux et al., 2017), prostate cancer (Grisanzio et al., 2012), and ovarian cancer (Fortner et al., 2017; Katchman et al., 2017; Kaaks et al., 2018)

Then risk scores calculated from m7G signatures were used to subdivide glioma patients into two risk groups with distinct immune microenvironments. We also created a m7G score using the “AddModuleScore” function of the “Seurat” R package to investigate the expression landscape of m7G regulators in a spatial expression dataset of GBM. It turned out that a cluster of cancer cells featured by high expression of PTN scored highest among the 7 clusters identified. These cells were embedded with SREBF1- and THY1-high expressing cancer cells. The SPP1 and PTN signaling pathways were then recognized by the “CellChat” R package as the most significant pathways of cell communication among these 3 cell types.

The secreted phosphoprotein 1 (SPP1) is an integrin-binding phosphorylated glycoprotein that has been reported to play important roles in several tumor-associated processes, including proliferation, invasion, migration, angiogenesis, and metastasis (Huang et al., 2017; Zeng et al., 2018; Chiou et al., 2019). In glioma, it is reported that tumor cells including glioma stem cells (GSCs) elaborate OPN into the local microenvironment where it acts as a chemokine for tumor-supportive monocytes and macrophages. OPN-mediated chemokine activity of macrophages depends on the interaction of OPN with integrin αvβ5 and CD44 (Zhu et al., 2004). The glioma infiltrating macrophages (GIMs) can also secrete OPN to further amplifies the recruitment of additional immune-suppressive monocyte and macrophages (Wei et al., 2018). Besides, OPN secreted by GIMs can also sustain glioma cell survival and stimulate angiogenesis (Chen et al., 2019). Moreover, OPN might also regulate the mesenchymal phenotype of glioma by interacting with CD44 on tumor cells (He et al., 2021).

The pleiotrophin (PTN) is a critical cytokine that regulates diverse physiological functions (Mitsiadis et al., 1995). PTN functions mainly through its receptor PTPRZ1 to increase phosphorylation of the downstream effectors, thereby activating the signal transduction related to cell growth, migration, and cellular activities (Meng et al., 2000; Kawachi et al., 2001; Deuel et al., 2002; Pariser et al., 2005). GIMs can secret abundant pleiotrophin (PTN) to stimulate glioma stem cells (GSCs) through its receptor PTPRZ1 thus promoting GBM malignant growth through PTN-PTPRZ1 paracrine signaling (Shi et al., 2017). Interestingly, our data suggested that cells with the highest m7G score also exhibited a high expression level of microglia/macrophage markers CD63 (Supplementary Table S6), and these cells were predicted to communicate with a cluster of cells with high expression of cancer stem cell maker THY1 via PTN pathway.

Our study illustrated that m7G modification was associated with glioma since most m7G-related genes were differentially expressed between normal and glioma tissues. Moreover, risk scores calculated from prognostic m7G signatures can predict outcomes of glioma patients. Our analyses also revealed that the tumor immune microenvironment was significantly different between the two risk groups stratified by risk scores in terms of HLA genes, immune checkpoint genes, the composition of infiltrated immune cells, and the activity of immune-related pathways. mRNA m7G modification is known to regulate RNA metabolism including alternative splicing. EIF4E1B was found to associate with OS-SEs most significantly among differentially expressed m7G regulators and we also reported the dual functions of this gene depending on glioma grade. Additionally, using spatial expression data, we found that the m7G score was highest in a cluster of cells that featured by high PTN level, which might communicate with adjacent tumor cells through SPP1 and PTN signaling pathways, further implying the regulatory role of m7G modification in the tumor-promoting microenvironment.

However, there are some limitations to be improved: Firstly, though we described a close connection in the expression of m7G regulators and a close correlation between m7G modification and TIMEs as well as ASEs, we failed to explain the mechanism behind these phenomena due to the lack of evidence in this filed currently. Secondly, in the section where independent prognostic factors were screened for constructing nomogram, only limited clinicopathological factors were included due to a considerable amount of missing data in the TCGA database, thus some potential prognostic factors (such as Karnofsky performance score, weight, and size of the tumor) might be omitted. Therefore, cohorts of glioma patients with transcriptomic data and well-documented clinical information are still needed to complement the current prediction model. Thirdly, we failed to find a cohort of glioma patients who share a similar genetic background to those in the TCGA cohort with transcriptomic data processed through a similar workflow to externally validate our result, thus there could be a high risk of selection bias. Studies should be carefully designed to further validate the current results. Last but not the least, this is a preliminary study whose results were derived from the analyses and interpretation of multi-omics data, thus experiments are needed to further support our findings.

In conclusion, our preliminary work provided novel insights into the relationship between m7G modification and the immune microenvironment of glioma as well as the regulatory role of m7G modification in alternative splicing in glioma, but further studies are needed to supplement and validate the current results.

## Data Availability

The datasets presented in this study can be found in online repositories. The names of the repository/repositories and accession number(s) can be found in the article/Supplementary Material.
