# Supplementary material for: A novel prognostic 7-methylguanosine signature reflects immune microenvironment and alternative splicing in glioma based on multi-omics analysis
Source: Front Cell Dev Biol. 2022 Aug 10;10:902394. doi: 10.3389/fcell.2022.902394 (PMC9399734; doi:10.3389/fcell.2022.902394)
Supplement: Supplementary file 3 [file DataSheet1.PDF]

Supplementary Figure 1 | The expression level of 33 m7G regulators were compared between normal tissue and GBM, normal tissue and LGG as well as GBM and LGG (Wilcox test, *p<0.05; **p<0.01; ***p<0.001)

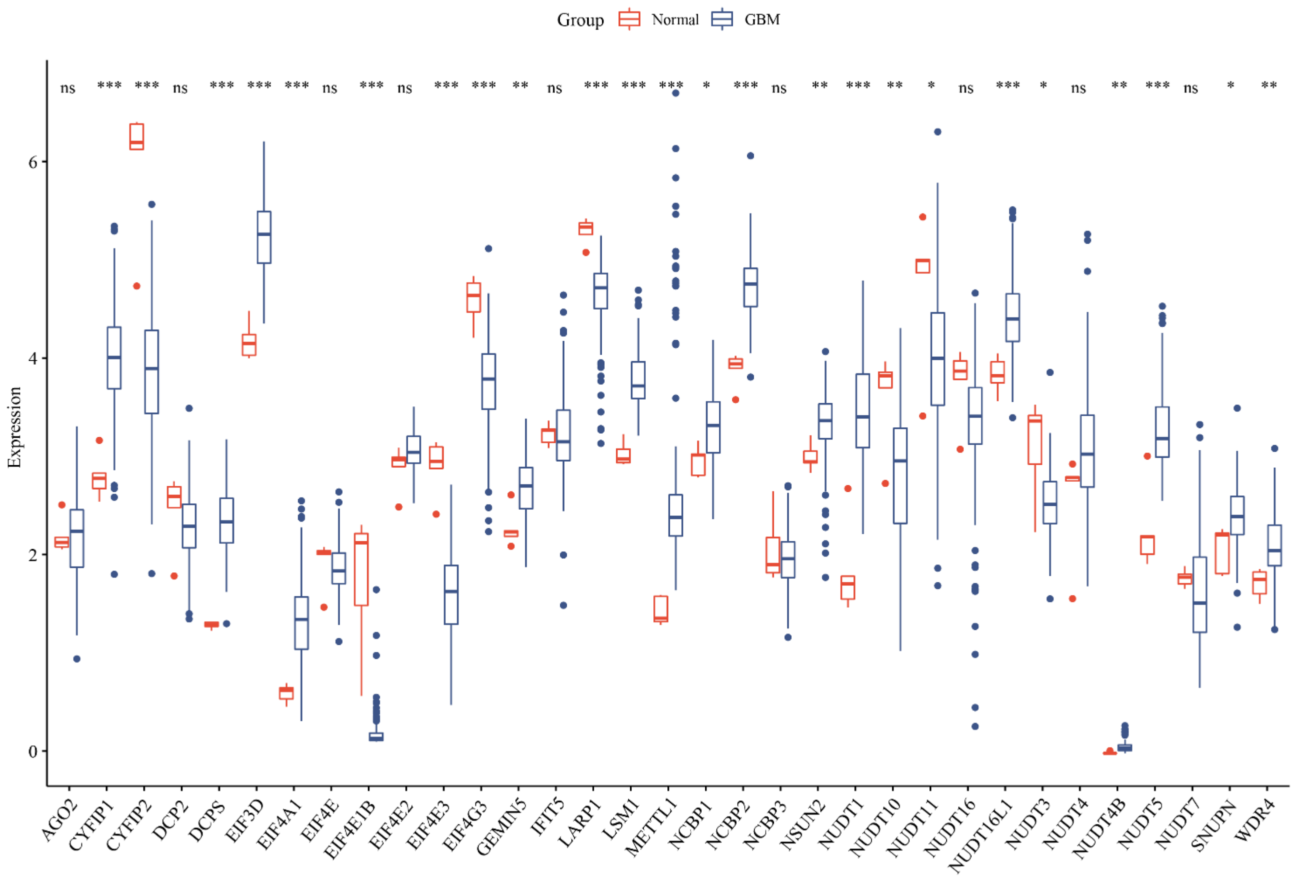

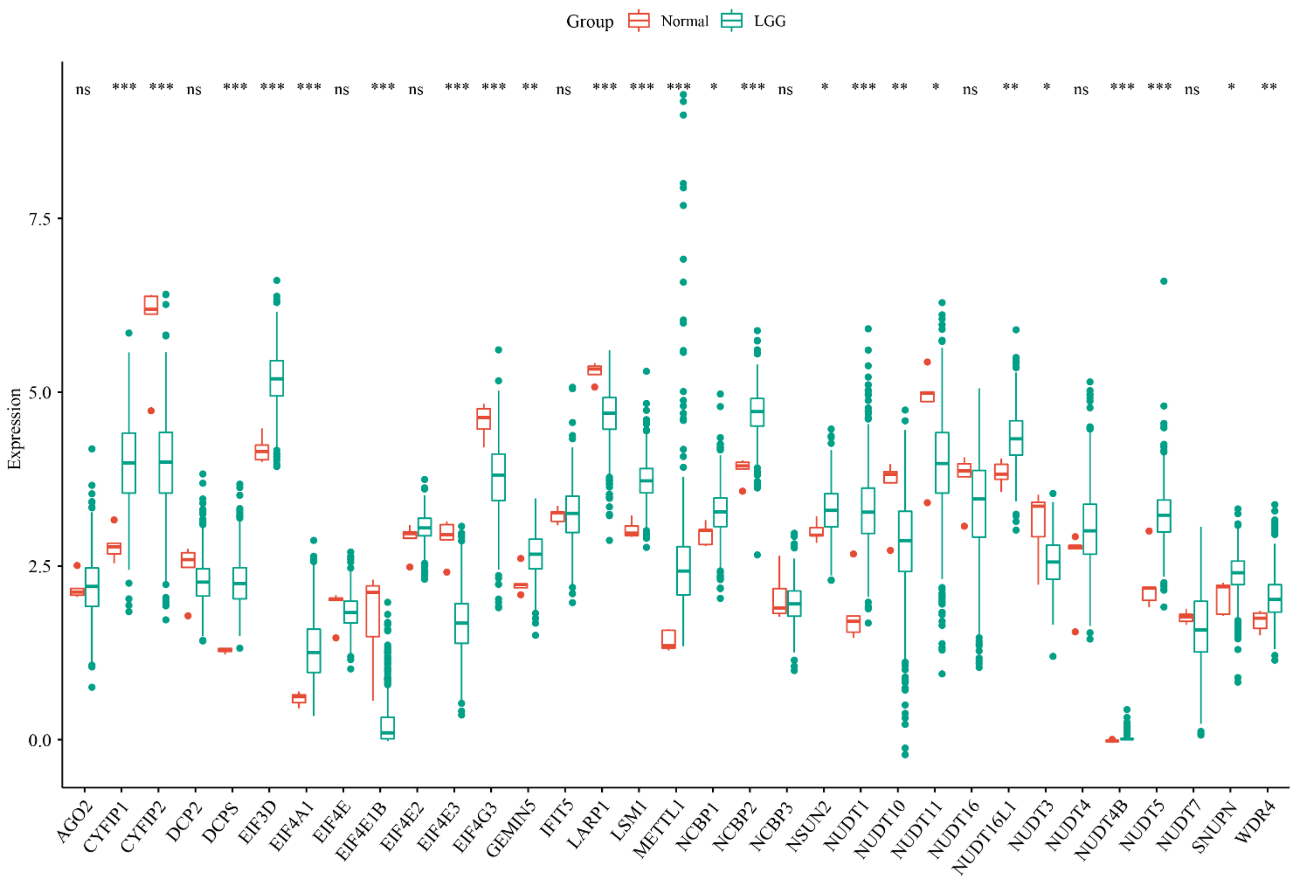

Group
Normal
LGG
Expression
0.0
2.5
5.0
7.5
ns *** *** ns *** *** *** ns *** ns *** *** ** ns *** *** *** * *** ns * *** ** * ns ** * ns *** *** ns * **
AGO2
CYFIP1
CYFIP2
DCP2
DCPS
EIF3D
EIF4A1
EIF4E
EIF4E1B
EIF4E2
EIF4E3
EIF4G3
GEMIN5
IFIT5
LARP1
LSM1
METTL1
NCBP1
NCBP2
NCBP3
NSUN2
NUDT1
NUDT10
NUDT11
NUDT16
NUDT16L1
NUDT3
NUDT4
NUDT4B
NUDT5
NUDT7
SNUPN
WDR4

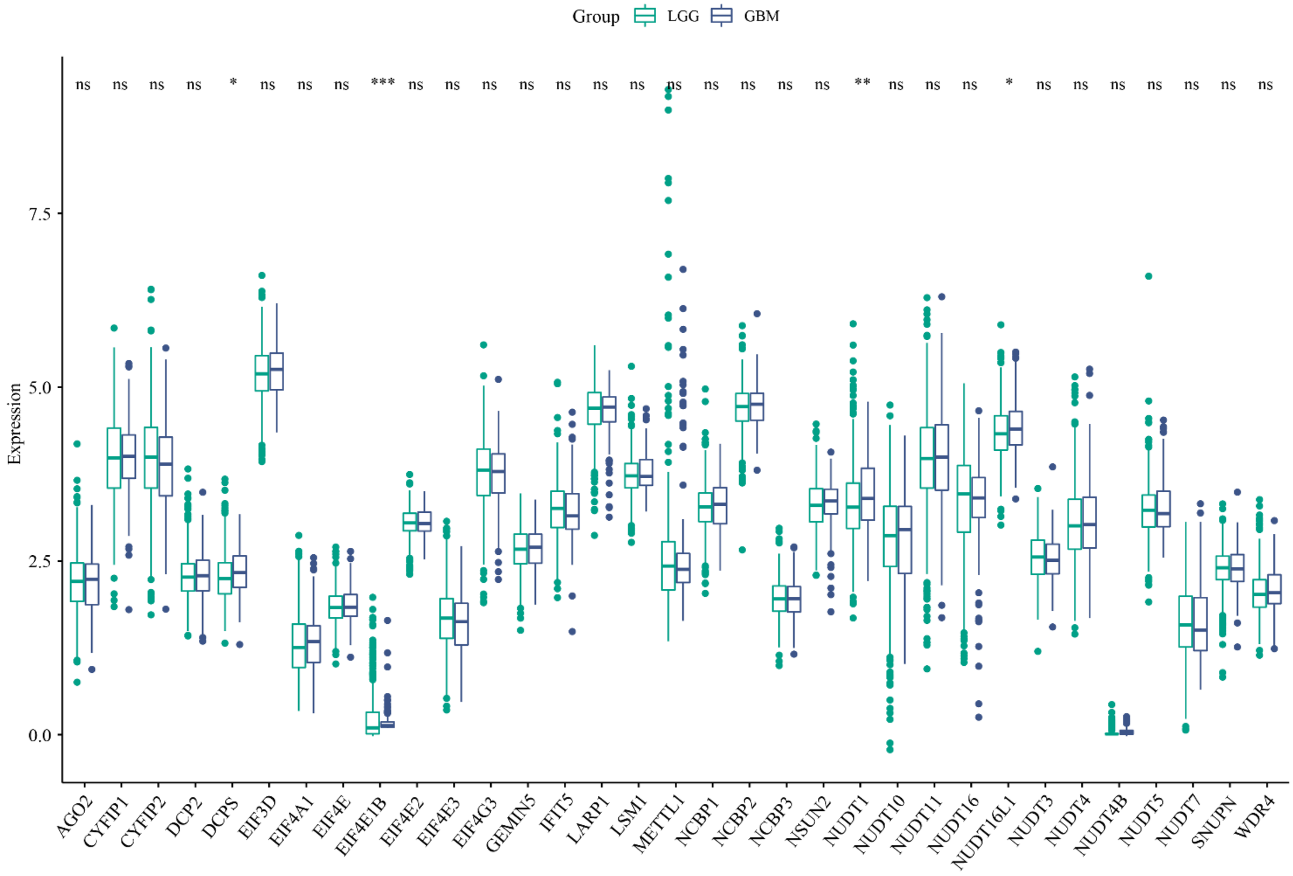
Group
LGG
GBM
Expression
0.0
2.5
5.0
7.5
ns ns ns ns * ns ns ns *** ns ns ns ns ns ns ns ns ns ns ns ns ** ns ns ns * ns ns ns ns ns ns ns
AGO2
CYFIP1
CYFIP2
DCP2
DCPS
EIF3D
EIF4A1
EIF4E
EIF4E1B
EIF4E2
EIF4E3
EIF4G3
GEMIN5
IFIT5
LARP1
LSM1
METTL1
NCBP1
NCBP2
NCBP3
NSUN2
NUDT1
NUDT10
NUDT11
NUDT16
NUDT16L1
NUDT3
NUDT4
NUDT4B
NUDT5
NUDT7
SNUPN
WDR4

Supplementary Figure 2 | mRNA and protein level of m7G signature genes

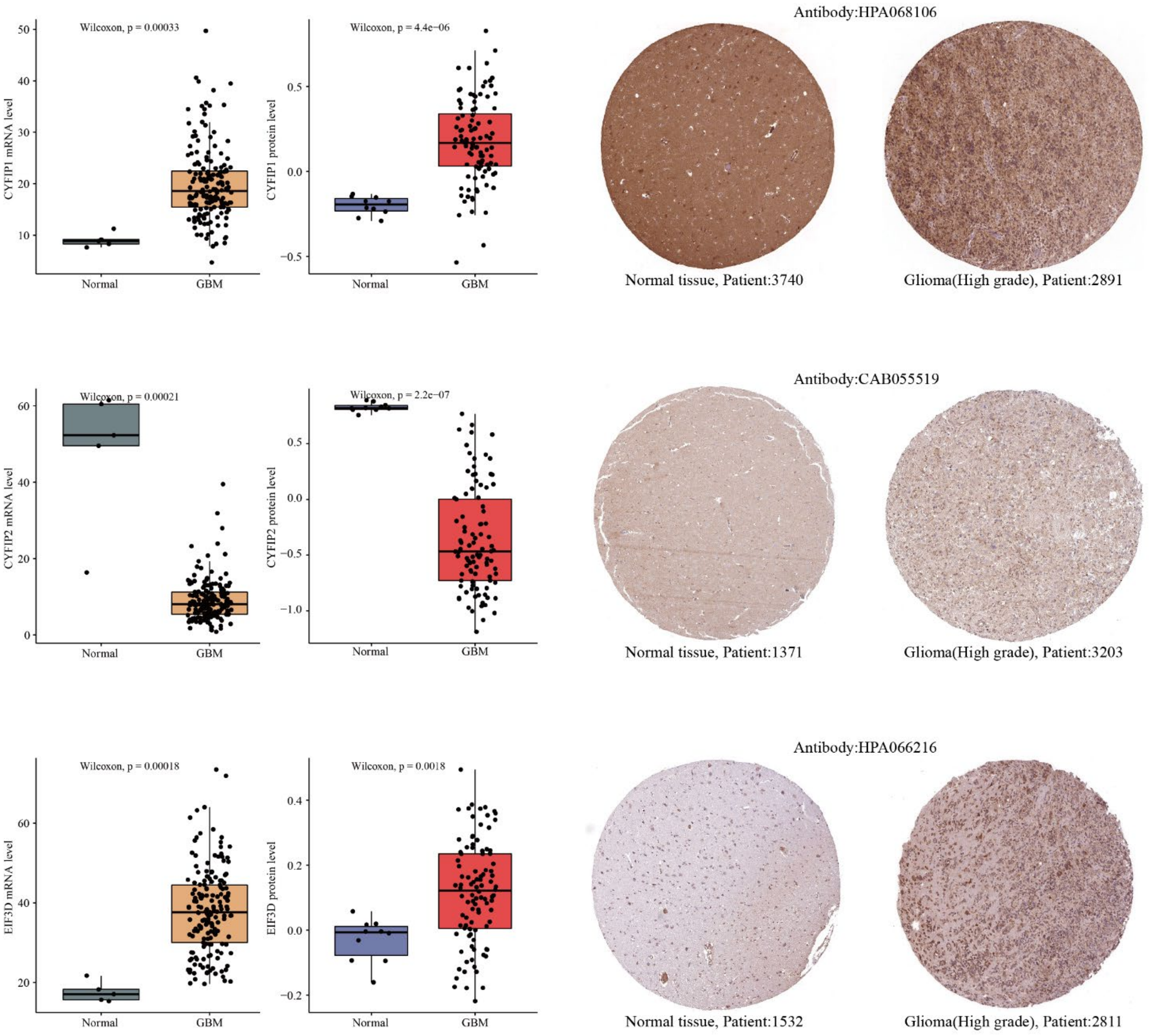

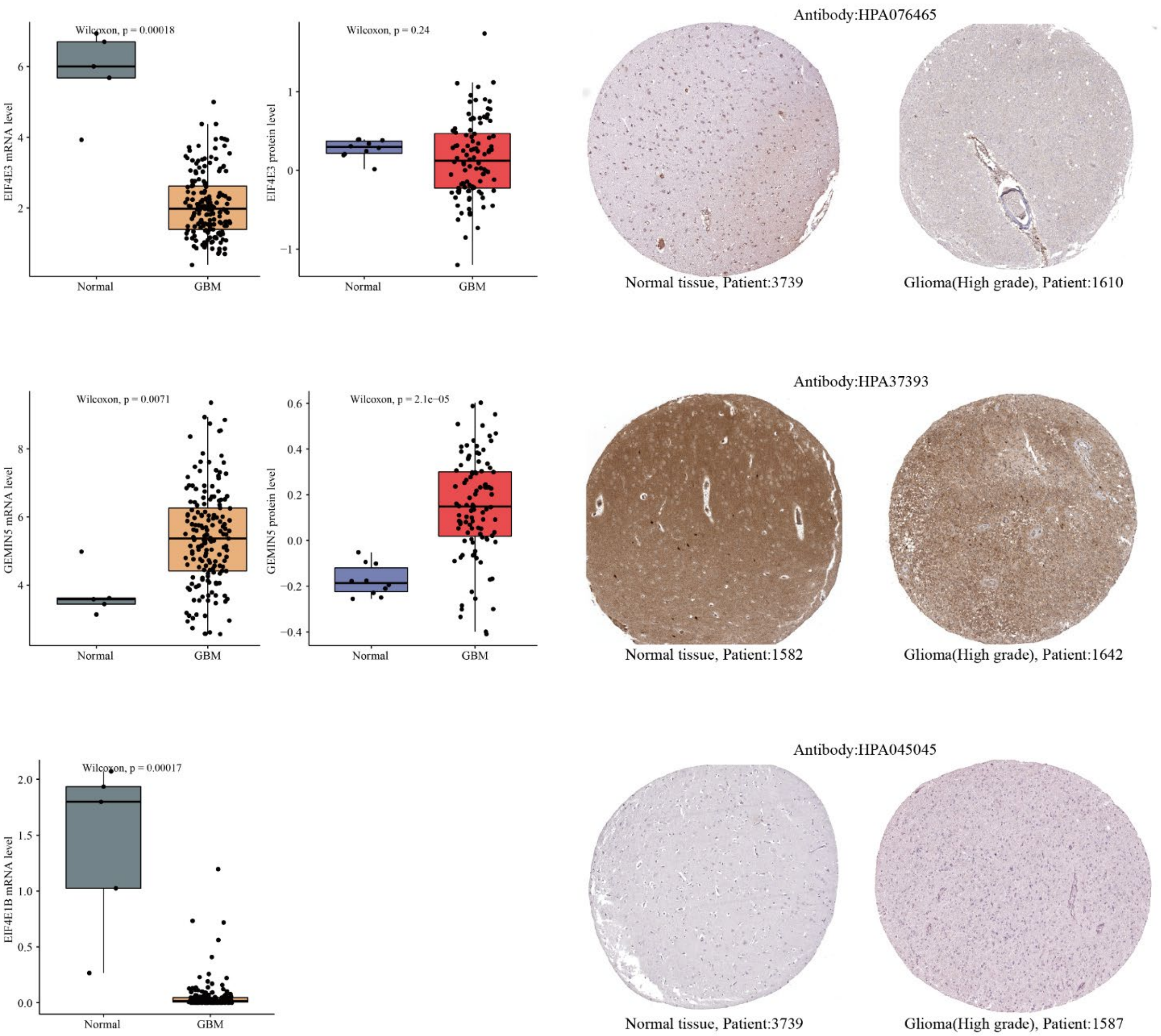
Wilcoxon, p = 0.00018
EIF4E3 mRNA level
Normal
GBM
Wilcoxon, p = 0.24
EIF4E3 protein level
Normal
GBM
Antibody:HPA076465
Normal tissue, Patient:3739
Glioma(High grade), Patient:1610
Wilcoxon, p = 0.0071
GEMIN5 mRNA level
Normal
GBM
Wilcoxon, p = 2.1e−05
GEMIN5 protein level
Normal
GBM
Antibody:HPA37393
Normal tissue, Patient:1582
Glioma(High grade), Patient:1642
Wilcoxon, p = 0.00017
EIF4E1B mRNA level
Normal
GBM
Antibody:HPA045045
Normal tissue, Patient:3739
Glioma(High grade), Patient:1587

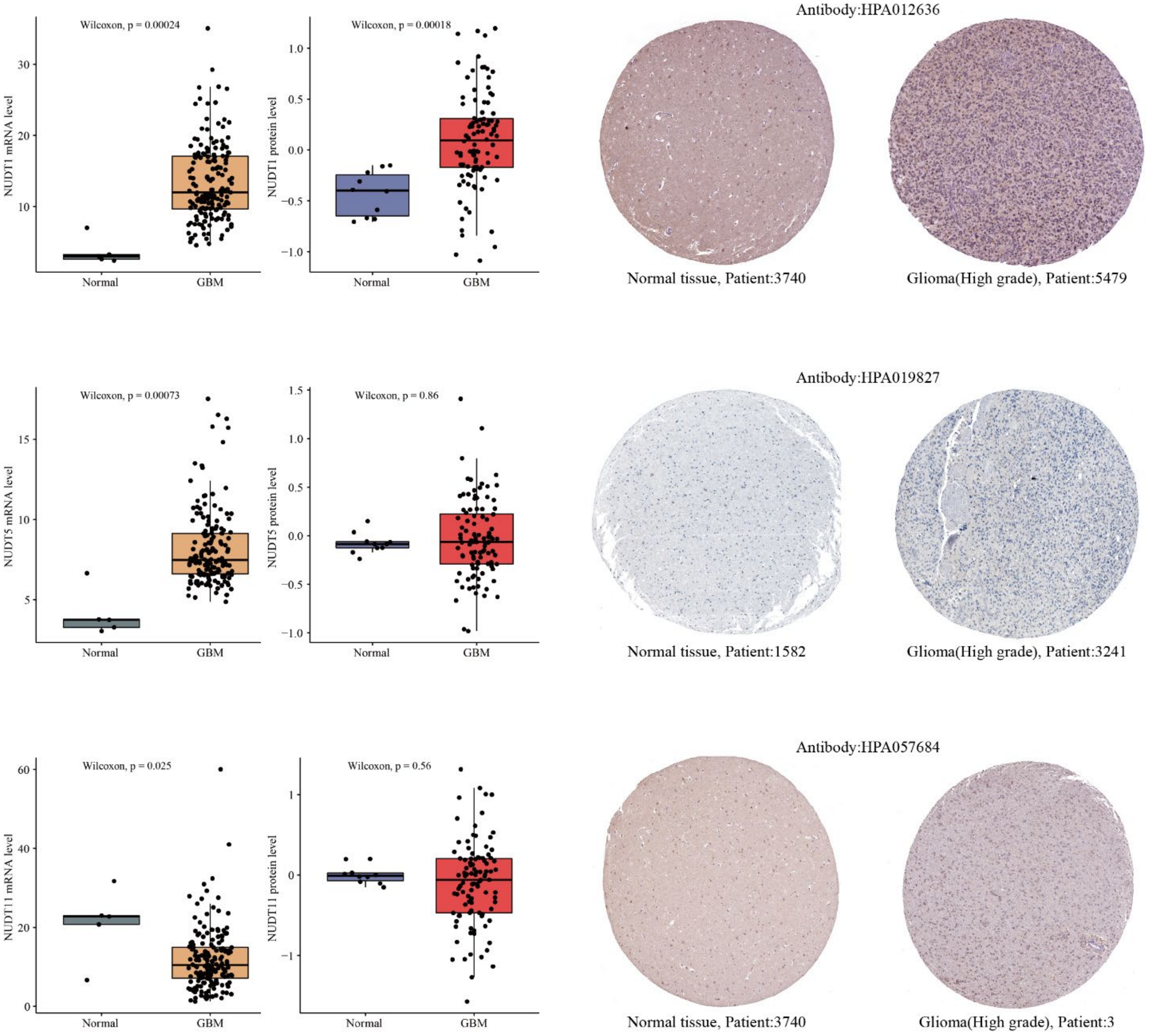
Wilcoxon, p = 0.00024
NUDT1 mRNA level
Normal
GBM
Wilcoxon, p = 0.00018
NUDT1 protein level
Normal
GBM
Antibody:HPA012636
Normal tissue, Patient:3740
Glioma(High grade), Patient:5479
Wilcoxon, p = 0.00073
NUDT5 mRNA level
Normal
GBM
Wilcoxon, p = 0.86
NUDT5 protein level
Normal
GBM
Antibody:HPA019827
Normal tissue, Patient:1582
Glioma(High grade), Patient:3241
Wilcoxon, p = 0.025
NUDT11 mRNA level
Normal
GBM
Wilcoxon, p = 0.56
NUDT11 protein level
Normal
GBM
Antibody:HPA057684
Normal tissue, Patient:3740
Glioma(High grade), Patient:3

Supplementary Figure 3 | Kaplan-Meier survival curve for the OS of patients subdivided by the infiltration level of immune cells in the GBM cohort **(A, B)** and LGG cohort **(C-H)** (yellow lines represent high infiltration groups and blue lines represent low infiltration groups)

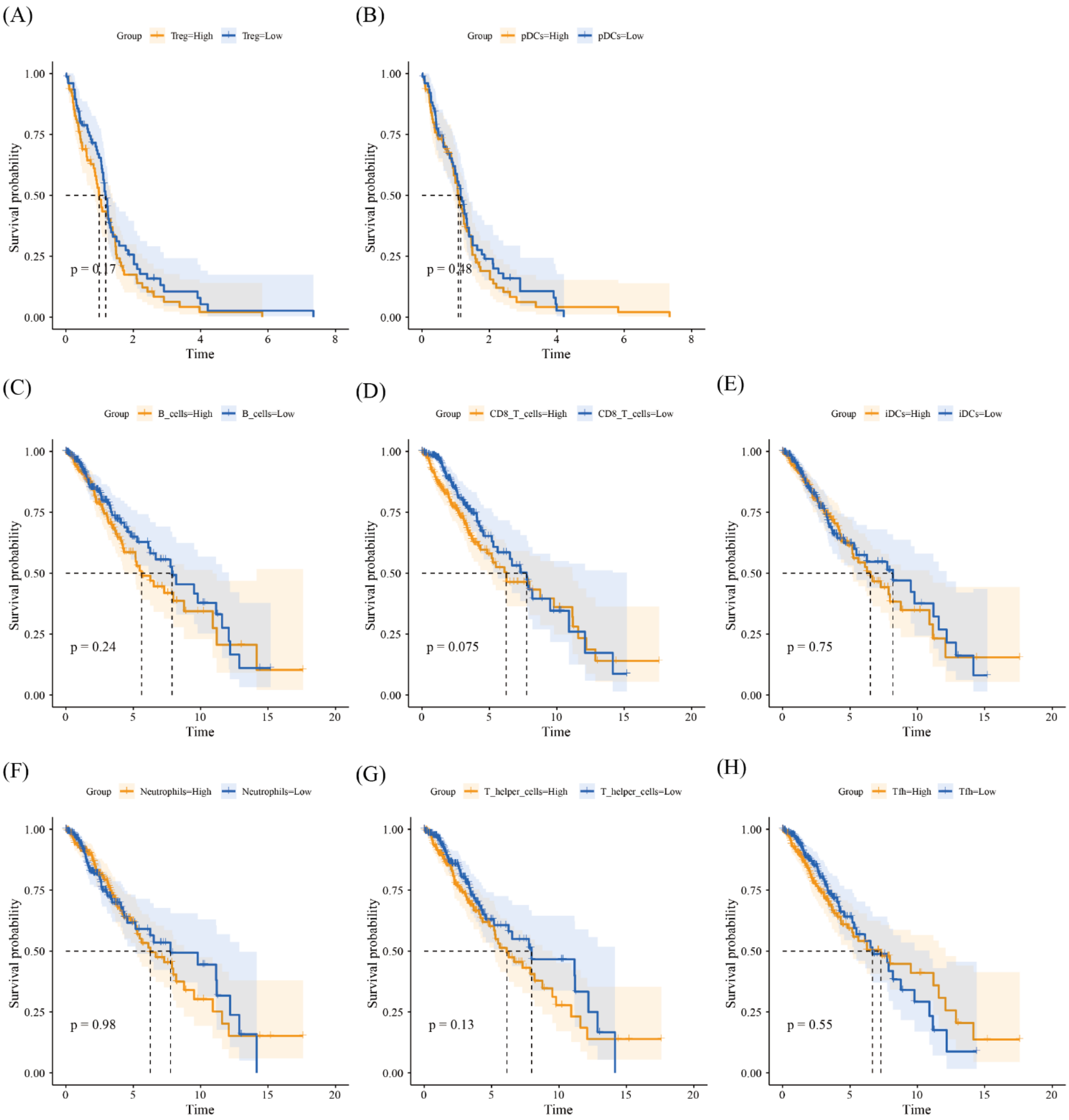

Supplementary Figure 4 | The volcano plot of prognosis-related ASEs in GBM (A). The bubble plot of the top 20 OS-ASEs in seven types of alternative splicing (B-H)

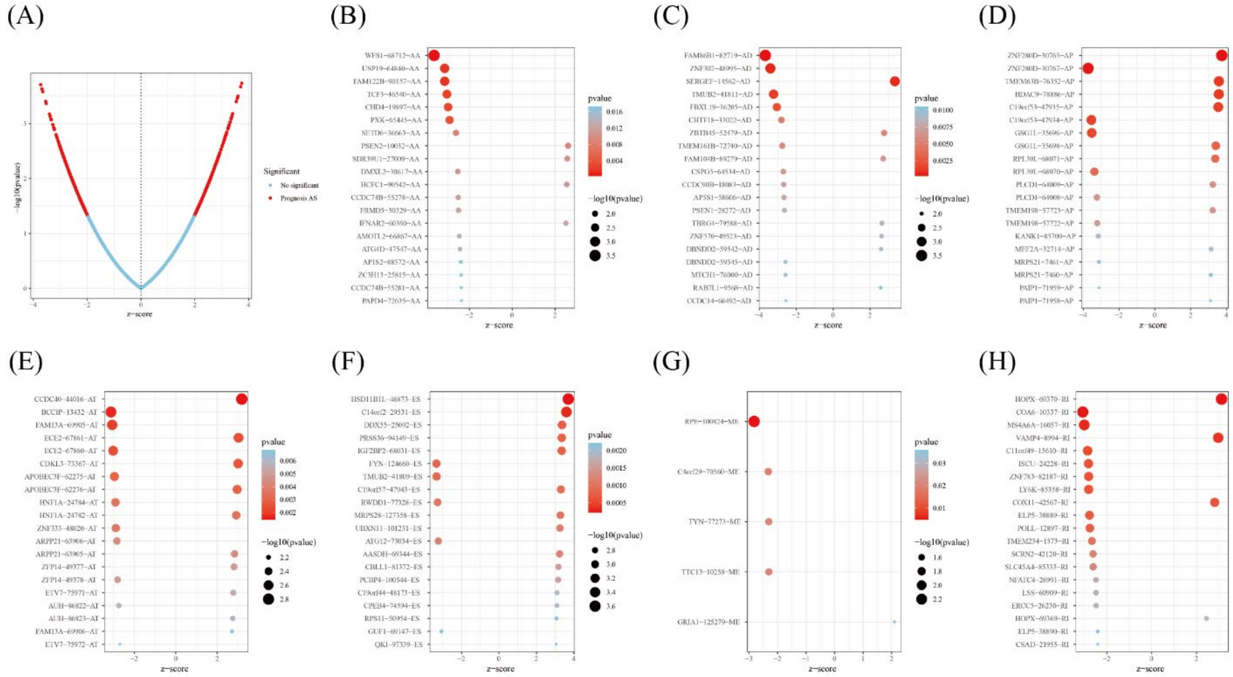

Supplementary Figure 5 | The volcano plot of prognosis-related ASEs in LGG (A). The bubble plot of the top 20 OS-ASEs in seven types of alternative splicing (B-H)

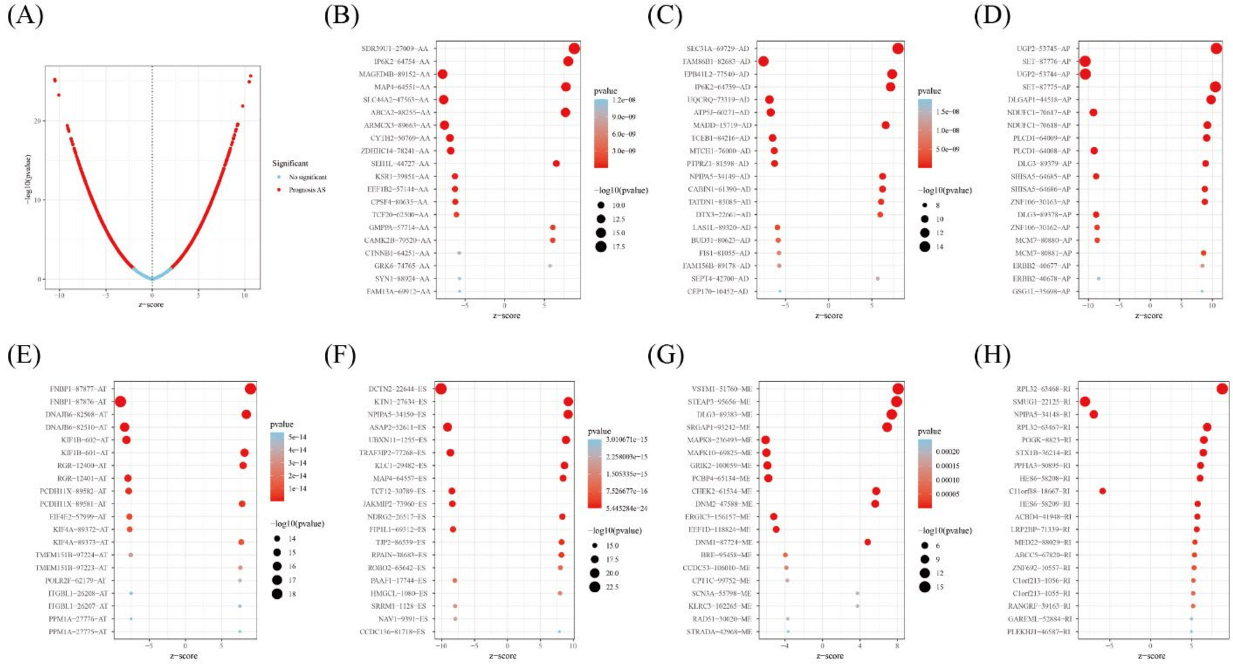

Supplementary Figure 6 | The splicing correlation network between m7G signature genes and OS-SEs in LGG (p<0.001 and |correlation coefficient|>0.6) was shown, where m7G signature genes were represented by yellow dots, risk ASEs with HR>1 were represented by red dots and favorable ASEs with HR<1 were represented by blue dots. Red arrows indicated positive correlations while blue arrows indicated negative correlations

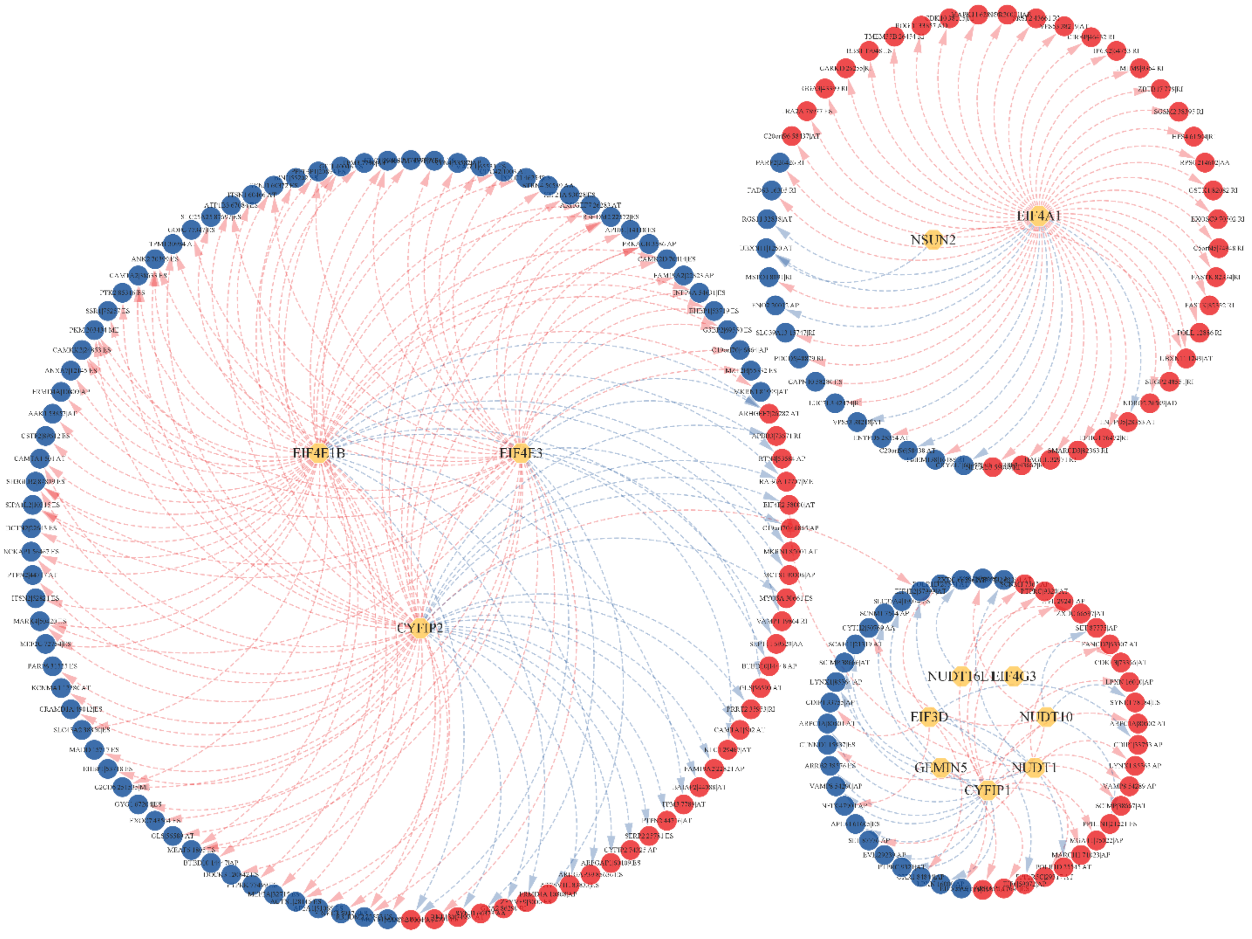

Supplementary Figure 7 | The cluster results of different resolutions determined by the "clustree" R package

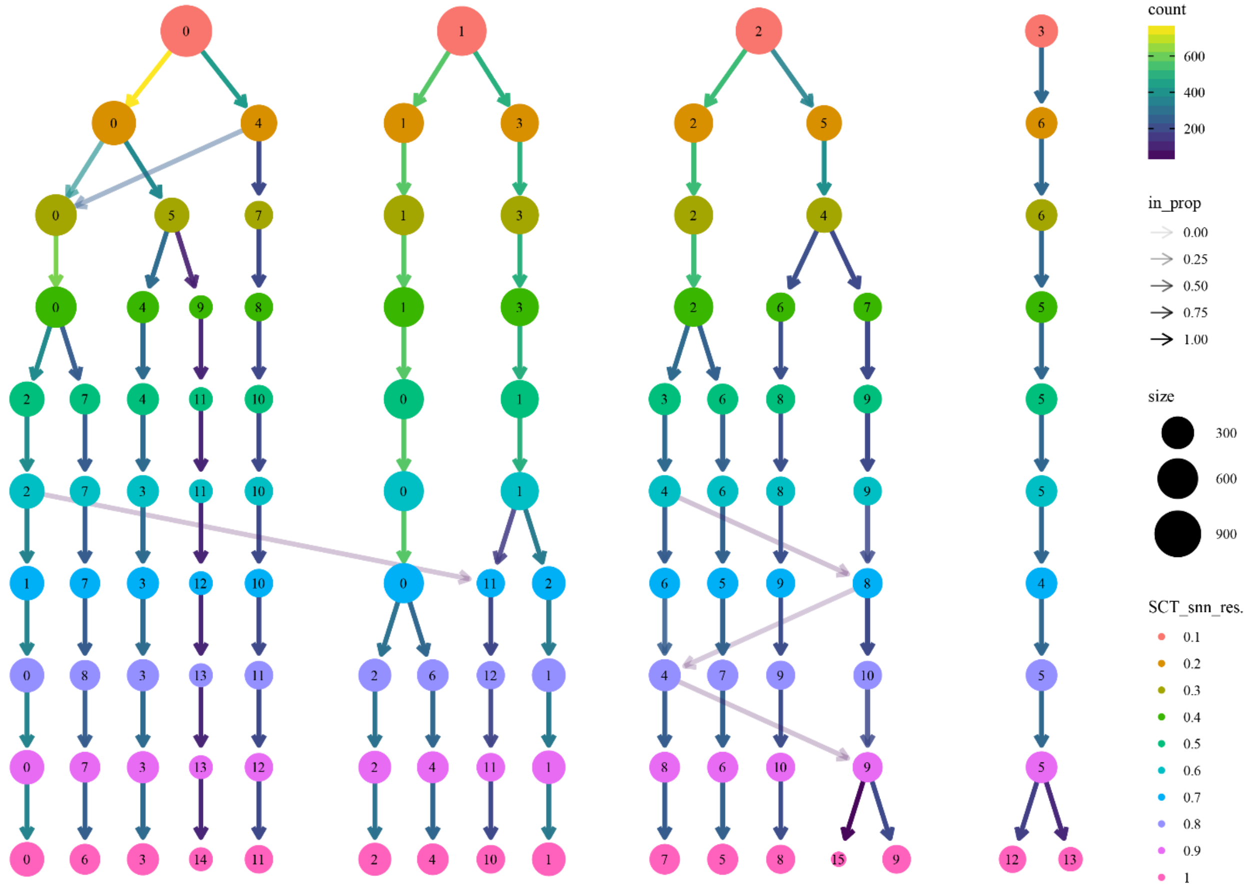

Figure S8 | A bubble plot showing the probability of cell communication among 3 clusters of cell type through known ligand-receptor pairs predicted by the “CellChat” R package.

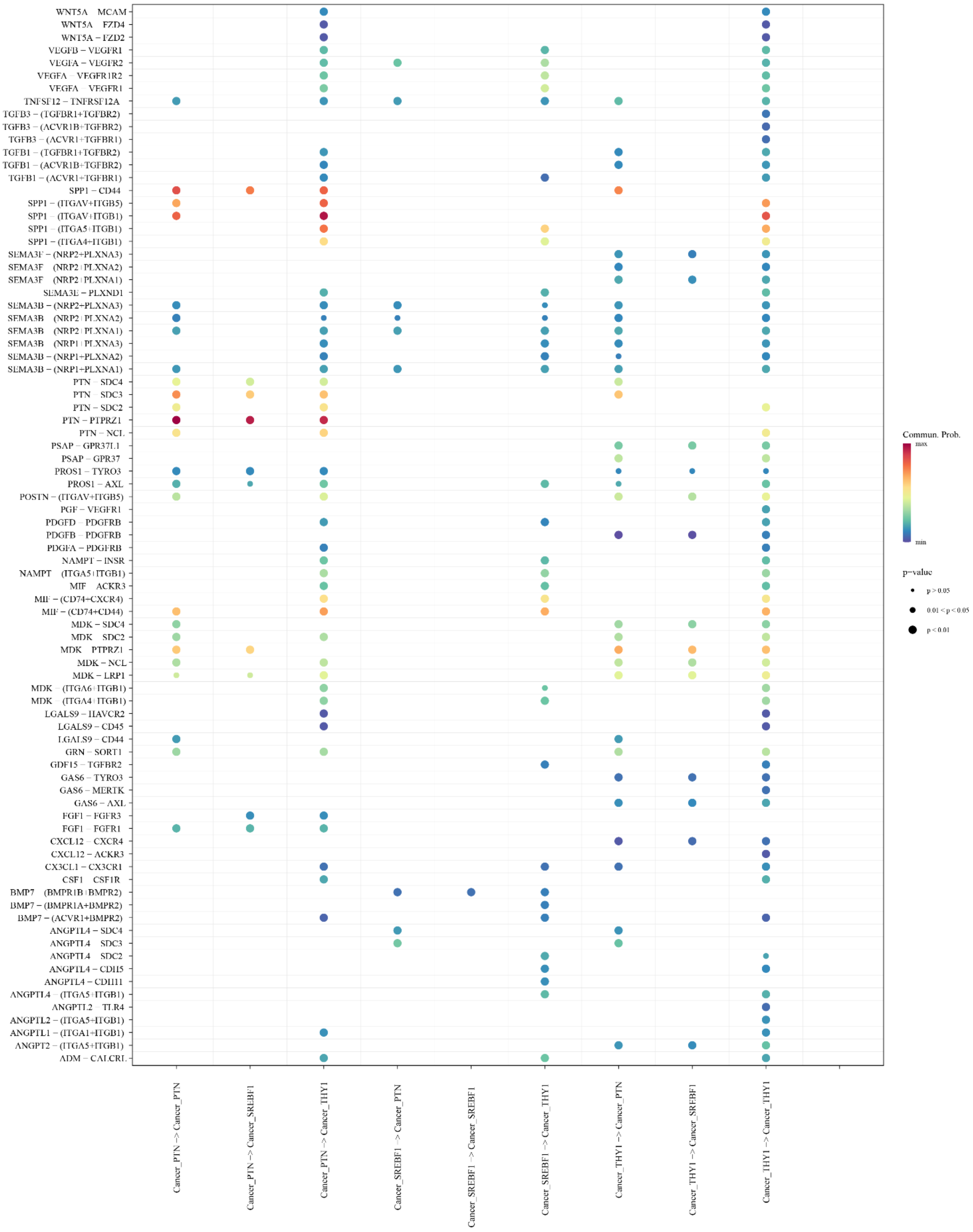

Supplementary Table 1: Table S1_m7G-related genes.xlsx

Supplementary Table 2: Correlation of m7G regulators with OS-SEs in GBM cohort

Supplementary Table 3: Correlation of m7G regulators and OS-SEs in LGG cohort.xlsx

Supplementary Table 4: Top10_cell_markers
